# Nrp1 Signaling Reprograms Glutathione Metabolism to Drive Mitochondrial Dysfunction in Severe Asthma

**DOI:** 10.3390/antiox15040463

**Published:** 2026-04-08

**Authors:** Junwen Huang, Wenqu Zhao, Ying Chen, Yaoxin Chen, Zhaoqian Gong, Yanyan Ma, Yuemao Li, Dapeng Hu, Shuyu Huang, Keke Fan, Bang Zhu, Xiaoqian Peng, Xianru Peng, Shaoxi Cai, Haijin Zhao

**Affiliations:** 1 Chronic Airways Diseases Laboratory, Department of Respiratory and Critical Care Medicine, Nanfang Hospital, Southern Medical University, Guangzhou 510515, China; 2 Department of Respiratory and Critical Care Medicine, Zhujiang Hospital, Southern Medical University, Guangzhou 510280, China

**Keywords:** severe asthma, glutathione metabolism, mitochondrial dysfunction, SLC25A39, Neuropilin-1

## Abstract

Mitochondrial dysfunction drives persistent inflammation in severe asthma, yet its upstream metabolic regulation remains unclear. Induced sputum from patients with severe asthma was analyzed and integrated with transcriptomic datasets from independent cohorts. Two mouse models (C57BL/6J) were used for in vivo validation with multi-omics profiling, and mechanistic studies were performed in air–liquid interface-cultured primary human airway epithelial cells. Glutathione reduced form (GSHr) was markedly depleted in sputum and associated with poor disease control and mixed granulocytic inflammation in patients with severe asthma. Multi-omics analyses revealed coordinated disruption of glutathione (GSH) metabolism, including oxidized GSH accumulation, reduced synthesis and glutathione-S-transferase activity, and impaired mitochondrial GSH transport. GSH supplementation alleviated airway inflammation, oxidative stress, and mitochondrial dysfunction, whereas pharmacological inhibition of GST exacerbated these effects. Mitochondrial analyses identified suppressed SLC25A39 expression as a key mediator of defective GSH transport and redox imbalance. Transcriptomic profiling of airway biopsies showed upregulation of Neuropilin-1 (Nrp1), closely associated with altered glutathione pathways. Targeting the Nrp1 b1 domain restored mitochondrial GSH metabolism and attenuated airway inflammation. These findings identify an Nrp-centered metabolic pathway that disrupts mitochondrial homeostasis and drives inflammatory amplification, highlighting mitochondria-targeted therapeutic strategies for severe asthma.

## 1. Introduction

Severe asthma remains a formidable challenge in global respiratory health. While biologic therapies have transformed the management of severe type 2 inflammatory asthma in recent years, the therapeutic landscape for other variants characterized by neutrophilic dominance continues to lag behind [1]. Our prior work established a toluene diisocyanate (TDI)-induced asthma model that recapitulated hallmark features of severe asthma, including steroid resistance, airway hyperresponsiveness (AHR), neutrophil-predominant airway inflammation, airway remodeling, and airway mitochondrial dysfunction [2,3]. This model provides a platform for investigating mechanisms that underlie therapeutic resistance in severe asthma, a critically unmet need in disease management [4].

Mitochondrial dysfunction is a hallmark of oxidative stress in chronic inflammatory diseases and has been increasingly recognized in asthma pathophysiology [5,6,7]. Central to these processes is glutathione (GSH), the most abundant intracellular non-protein thiol and a principal antioxidant, which plays a critical role in maintaining cellular redox homeostasis and cellular function [8]. GSH metabolism regulates redox balance through its de novo synthesis, enzymatic conjugation, and compartmentalized transport, particularly into mitochondria, where it buffers reactive oxygen species and preserves mitochondrial function [9,10,11]. The mitochondrial GSH carrier SLC25A39 is essential for sustaining antioxidant capacity and mitochondrial integrity, yet its role in airway inflammatory diseases remains unclear.

Neuropilin-1 (Nrp1) is a transmembrane co-receptor with multifaceted roles in immunity and cellular signaling [12]. It has emerged as a potential regulator of asthma pathogenesis. Structurally, Nrp1 comprises three extracellular domains: a1/a2, b1/b2, and c, each domain engaging distinct ligands to modulate disease processes [13]. For example, the a1/a2 domain binds semaphorin-3A, a molecule that is reduced in asthmatic serum and exhibits anti-inflammatory properties [13]. Conversely, the b1/b2 domain interacts with pro-fibrotic and pro-angiogenic factors such as TGF-β and VEGFA [13], which are both implicated in airway remodeling and proinflammatory responses in asthma [14]. These domain-specific dichotomies position Nrp1 as a compelling therapeutic target for asthma, although its role in asthma pathogenesis remains underexplored.

Based on prior research indicating Nrp1’s potential connection to redox pathways [15,16], we hypothesized that Nrp1 signaling disrupts GSH metabolism, leading to epithelial oxidative injury and inflammation. Therefore, this study aimed to define the functional role of Nrp1’s b1 domain signaling in severe asthma, elucidate its impact on GSH metabolism and mitochondrial redox balance, and evaluate the therapeutic potential of selectively targeting Nrp1’s b1 domain in experimental models of severe asthma.

## 2. Materials and Methods

### 2.1. Clinical Trial

Participants were recruited from the respiratory clinic of Nanfang Hospital. The hospital’s Ethics Committee granted approval for this study under approval number NFEC-2023-259. In total, 91 individuals were progressively included in the trial after receiving an asthma diagnosis in accordance with the Global Initiative for Asthma (GINA) 2024 guidelines. Respiratory tract infections and respiratory disease flare-ups within 4 weeks of the initial research visit were both grounds for exclusion. Severe asthma patients were classified according to GINA guidelines and belonged to GINA steps 4–5. As controls, 30 healthy individuals without a history of lung conditions were included with normal spirometry results. Standardized processes were used to induce and process sputum as described in our previous study [17]. Informed consent was obtained from all subjects, and the experiments conformed to the principles set out in the WMA Declaration of Helsinki and the Department of Health and Human Services Belmont Report. Clinical trial number: not applicable (this is a non-interventional observational clinical cohort).

### 2.2. Animal

Male C57BL/6J mice (6-week-old) were obtained from Southern Medical University. All mice were kept in a specific pathogen-free (SPF) laboratory with a 12 h dark/light cycle at a temperature of 23 ± 2 °C and a humidity range of 40–70%. Mice were continuously given irradiated food and sterilized water. All mice were randomly housed in separate cages, and interventions and analyses were conducted at the same time point.

### 2.3. Establishment of the TDI-Induced Mice Asthma Model

A total of 90 mice were randomly allocated to 9 groups by simple randomization. A mixture of 2 vol of acetone and 3 vol of olive oil was used as the vehicle to dissolve TDI for cutaneous sensitization, and 1 vol of acetone and 4 vol of olive oil was utilized for airway challenge. The procedure of establishing animal models was described in our previous report [3]. ICG-001 (5 mg/kg, i.p.), a selective inhibitor of the β-catenin/CBP interaction; EG00229 (5 mg/kg, i.p.), a Nrp1-b1 domain antagonist; olopatadine (5 mg/kg, i.p.), a histamine H1 receptor antagonist; dabrafenib (5 mg/kg, i.p.), a selective BRAF kinase inhibitor; EG01377 dihydrochloride (5 mg/kg, i.p.), an inhibitor of a1 and b1 domains of Nrp1; GSTO-IN-2 (10 mg/kg, i.p.), a glutathione S-transferase inhibitor; and reduced glutathione (GSHr, 100 mg/kg, i.p.), an antioxidant that replenishes intracellular glutathione levels, were dissolved in DMSO and saline and administered to mice prior to each sensitization and airway challenge. The dosage of drugs in vivo and in vitro was referenced from previous research [2,15,18,19,20,21,22,23,24,25,26,27]. Sham mice were given the same amount of vehicle.

### 2.4. Establishment of the HDM/LPS-Induced Mice Asthma Model

A total of 60 mice were randomized to 6 groups. Mice were sensitized and challenged with 20 μg HDM i.n. on day 0, day 7, days 14–18, and days 21–25. LPS (10 μg/mouse) was administered intratracheally on day 21. The mice in the control group were sensitized and challenged by inhaling saline. Glutathione reduced form (100 mg/kg, i.p.), EG00229 (5 mg/kg, i.p.), olopatadine (5 mg/kg, i.p.), and dabrafenib (5 mg/kg, i.p.) dissolved in DMSO and saline were given to the mice immediately before each sensitization and airway challenge.

### 2.5. Preparation of TDI–Human Serum Albumin (HSA) Conjugates

In accordance with our reported procedure [28], TDI–human serum albumin (HSA) conjugates were prepared. Phosphate-buffered saline (PBS) was used as the vehicle to dissolve TDI-HSA conjugates. Sham cells were given the same amount of vehicle.

### 2.6. Cell Culture and Treatment

Primary human airway epithelial cells (HAECs) were obtained from ATCC. They were expanded in PneumaCult™-Ex Plus Medium. Once reaching 85% convergence in tissue culture flasks (NEST Science Co., Ltd., Shanghai, China), the cells were passaged and seeded into suitable culture plates at a density of 1 × 10^5^ cells/cm^2^. The dispersed cells were then passaged at the same plating density into further T25 flasks or seeded on apical chambers of 12 mm Transwell (0.4 µm Pore Polyester Membrane Inserts, Costar, Corning, NY, USA). Within the inserts, cells were incubated at 37 °C until 100% confluence was reached (2~4 days). Upon reaching confluence, the medium for expansion was removed from the inserts and washed with D-PBS. Then the PneumaCult™-ALI Maintenance Medium was added to the basal chamber only, leaving the apical chamber empty, to create an air–liquid interface (ALI) for the cells. The PneumaCult-ALI protocol for the maintenance phase was then followed for 28 days. Cells within five passages were used for our experiments. Cells were harvested for protein extraction, and supernatant in the basal chamber was collected 24 h post-exposure. A test for mycoplasma contamination should be performed prior to each cell repassaging and sample harvesting to ensure the absence of contamination.

### 2.7. Sample Size Calculation

Sample size was determined based on previous similar studies [3] and power analysis to detect significant differences in airway inflammation. The final sample size will be larger than the number required for calculation to meet different experimental needs.

### 2.8. Blinding

Group allocation was concealed during the allocation process and the conduct of the experiment. Investigators responsible for outcome assessment and data analysis were blinded to the group assignments to minimize bias.

### 2.9. Assessment of Airway Hyperresponsiveness (AHR)

A chamber (Buxco Electronics, Troy, NY, USA) was employed to measure each mouse’s airway responsiveness to methacholine on day 25. To achieve this, lung resistance (RL) was gauged in response to escalating dosages (6.25, 12.5, 25, and 50 mg/mL) of methacholine through ultrasonic nebulization on anesthetized and mechanically ventilated mice. After every nebulization process, measurements of RL were taken at 5 min intervals until a plateau phase was attained. The results were presented as a percentage of the RL value at baseline for each methacholine concentration.

### 2.10. Quantification of Total Serum IgE

After resting at room temperature for an hour, the blood samples were centrifuged (3000× *g*, 20 min), and the supernatant fluid was collected and kept at −80 °C. Following the manufacturer’s instructions, we evaluated total serum IgE via an ELISA kit for IgE.

### 2.11. Analysis of Bronchoalveolar Lavage (BAL) Fluids

The number of cells in BAL fluid was calculated via a cell counter (Count-star, Shanghai, China). Then, the BAL fluid was centrifuged (1000× *g*, 10 min). According to the manufacturer’s recommendations, the supernatant was frozen (−80 °C) for subsequent IL-33, IL-4, IL-5, IL-17, and IL-13 ELISA analysis. To classify different cells, cytospin samples were made from cell precipitation after centrifugation and stained using hematoxylin and eosin (H&E). A total of 200 cells from each sample were counted to determine the number of macrophages, eosinophils, neutrophils, and lymphocytes.

### 2.12. Pulmonary Histopathological Examination

The left lungs of the mice were separated, fixed with 4% paraformaldehyde, then dehydrated and paraffin-embedded. Lung tissue sections (4 μm) were cut and stained with periodic acid–Schiff (PAS), H&E, and Masson’s trichrome. Briefly, two criteria were scored to document the pulmonary inflammation: peribronchial inflammation and perivascular inflammation. A value of 0 was adjudged when no inflammation was detectable, a value of 1 for occasional cuffing with inflammatory cells, a value of 2 for most bronchi or vessels surrounded by thin layer (one to ten cells) of inflammatory cells, and a value of 3 when most bronchi or vessels were surrounded by a thick layer (more than ten cells) of inflammatory cells. As 8–10 tissue sections per mouse were scored, inflammation scores could be expressed as a mean value and could be compared between groups. Sections were assigned a random code to blind the examiner to the identity of each specimen.

### 2.13. Transmission Electron Microscopy

We used 1.25% glutaraldehyde/0.1 M phosphate buffer to fix lung tissue. An electron microscope (JEOL, JEM-1010, Tokyo, Japan) was used to examine ultrathin sections (60 nm), which were prepared by a routine procedure.

### 2.14. Co-Immunoprecipitation

Thermo Scientific Pierce^TM^ Classic Magnetic IP/CO-IP Kit (Waltham, MA, USA) was used in accordance with the manufacturer’s instructions to perform the coimmunoprecipitation between Nrp1 and VEGFA. Briefly, antibodies were covalently crosslinked to Protein A/G beads using dimethyl pimelimidate to avoid interference from antibody heavy and light chains. Briefly, antibodies were first incubated with prewashed Protein A/G beads in PBS for 30–60 min at 4 °C with rotation, followed by washing with 0.2 M triethanolamine (pH 8.3). Freshly prepared dimethyl pimelimidate (20 mM in 0.2 M triethanolamine) was then added, and the mixture was incubated for 30 min at room temperature. Unreacted sites were quenched with 0.2 M ethanolamine (pH 8.0) for 15 min, and the beads were extensively washed with PBS or IP buffer. The crosslinked beads were used immediately for immunoprecipitation or stored at 4 °C in PBS containing 0.02% sodium azide.

### 2.15. Mitochondria Isolation

Tissue and cell mitochondria isolation was performed according to the manufacturer’s protocol (Beyotime, Shanghai, China).

### 2.16. Quantitative PCR (qPCR)

In accordance with the manufacturer’s instructions, total RNA was extracted with the use of the Total RNA Extraction Reagent. Using 2 μg of RNA in a 20 μL reaction buffer of HiScript^®^ IV All-in-one RT SuperMix (Vazyme, Nanjing, China), perfect for qPCR kits and random primers, the first strand of complementary DNA was generated by sequentially heating the mixture to 37 °C for 15 min and 85 °C for 5 s. Real-time PCR was performed using a ChamQ Universal SYBR qPCR Master Mix (Vazyme, Nanjing, China).

### 2.17. Detection of GSH Metabolism

The detection of GSH metabolism was performed at the GSH Assay Kit (ABclonal Technology, Wuhan, China), GSS Assay Kit (Norminkoda/Bionmkd, Wuhan, China), and GST Assay Kit (Bioesn, Shanghai, China) in accordance with the manufacturer’s protocol. Mitochondria were extracted from mouse lung tissue using a Tissue Mitochondria Isolation Kit (Beyotime, Shanghai, China).

### 2.18. Detection of Cell Viability and Mitochondrial Oxidative Stress

Cell Counting Kit-8, lactate dehydrogenase (LDH), and MitoSOX Red Mitochondrial Superoxide Indicator were utilized for the detection of cell viability and mitochondrial oxidative stress in HAECs in accordance with the manufacturer’s protocol.

### 2.19. Measurements of Mitochondrial Oxygen Consumption Rate

The rates of mitochondrial oxygen consumption in HAECs were measured in accordance with the manufacturer’s protocol (Seahorse Bioscience, Agilent Technologies, Santa Clara, CA, USA).

### 2.20. Detection of Cell Permeability

Cell permeability was detected by FITC-labeled dextran (Beyotime, Shanghai, China) according to the manufacturer’s protocols.

### 2.21. Measurement of Oxidative Stress Levels

Total antioxidant capacity (TAC) and malondialdehyde (MDA) were assayed using the TAC assay kit (Thermo Scientific, Waltham, MA, USA) and the MDA assay kit (Beyotime, Shanghai, China), respectively, according to the manufacturer’s instructions.

### 2.22. Transcriptomics

Total RNA was extracted from lung tissue using the Accuracode cracking mixture reagent (Singleron, Nanjing, China). RNA integrity was assessed with the Agilent Bioanalyzer 2100 (Santa Clara, CA, USA). Sequencing libraries were prepared using the Illumina TruSeq kit (San Diego, CA, USA) and sequenced on the Illumina platform. Differentially expressed genes were identified using DESeq2 version 1.44.0 with adjusted *p* < 0.05 as the cutoff. Data was stored in the BioStudies database (S-BSST1232).

### 2.23. Metabolomics

Lung tissue samples were subjected to untargeted metabolomic profiling using UPLC-MS/MS. For two-group analysis, differential metabolites were determined by VIP (VIP > 1) and *p*-value (*p*-value < 0.05, Student’s *t* test). For multi-group analysis, differential metabolites were determined by VIP (VIP > 1) and *p*-value (*p*-value < 0.05, ANOVA). VIP values were extracted from the OPLS-DA result, which also contains score plots and permutation plots, and was generated using the R package MetaboAnalystR version 4.0. The data were log-transformed (log2) and mean-centered before OPLS-DA. In order to avoid overfitting, a permutation test (200 permutations) was performed. Identified metabolites were annotated using the KEGG Compound database, and annotated metabolites were then mapped to the KEGG Pathway database.

### 2.24. Statistical Analysis

Statistical analyses were performed using SPSS version 26.0. The normality of data distribution was assessed using the Shapiro–Wilk test, and homogeneity of variance was evaluated by Levene’s test. Normally distributed data were presented as the mean ± standard error (SE), while non-normally distributed data were expressed as the median (interquartile range, IQR). For comparisons between two independent groups, Student’s *t*-test was used for normally distributed data, and the Mann–Whitney U test was applied for non-normally distributed data. For comparisons among three or more groups, one-way analysis of variance (ANOVA) followed by Bonferroni post hoc test was used for normally distributed data with equal variances; otherwise, Welch’s ANOVA or the Kruskal–Wallis test followed by Dunn’s multiple comparisons test was performed as appropriate. Correlation analyses were conducted using Pearson’s correlation for normally distributed data and Spearman’s rank correlation for non-normally distributed data. A two-tailed *p*-value < 0.05 was considered statistically significant.

## 3. Results

### 3.1. Phenotype-Specific Alterations in Sputum Glutathione Reduced Form (GSHr) Reflect Asthma Severity

**Figure 1:**
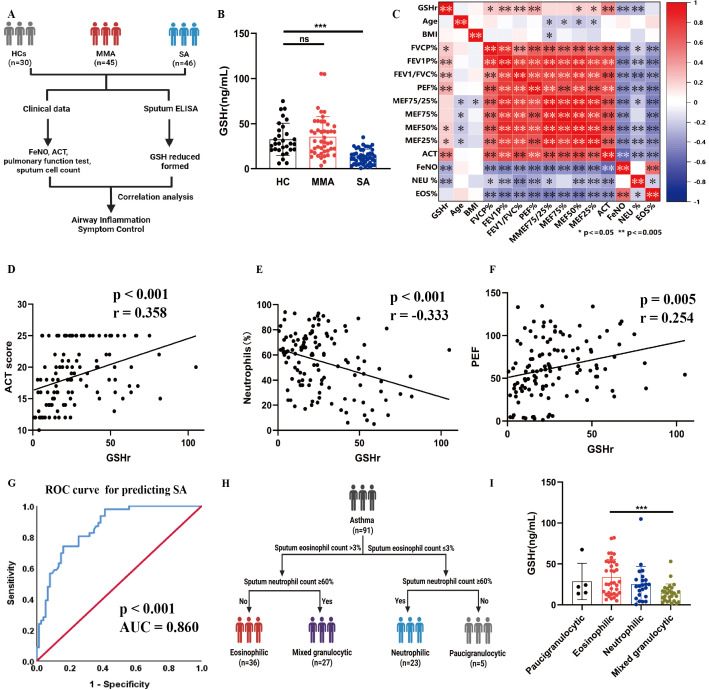
Phenotype-specific alterations in sputum GSHr reflect asthma severity. (**A**) Schematic of a clinical trial. (**B**) Levels of GSHr in healthy control subjects (HCs, n = 30), mild-to-moderate asthma (MMA, n = 45) patients, and severe asthma (SA, n = 46) patients. (**C**) Correlation analysis between sputum GSHr and clinical features in all subjects. (**D**) Correlation analysis between sputum GSHr and ACT score. (**E**) Correlation analysis between sputum GSHr and sputum neutrophil counts. (**F**) Correlation analysis between sputum GSHr and PFE. (**G**) ROC curve for sputum GSHr in predicting the diagnosis of severe asthma. (**H**) Schematic of subgroup analysis. (**I**) Levels of GSHr in paucigranulocytic asthma (n = 5), eosinophilic asthma (n = 36), neutrophilic asthma (n = 23), and mixed granulocytic asthma (n = 27). * *p* < 0.05, ** *p* < 0.005, *** *p* < 0.0005, ns > 0.05.

We recruited 30 healthy controls (HCs), 45 patients with mild-to-moderate asthma (MMA), and 46 patients with severe asthma (SA) (Figure 1A). Table 1 presents the characteristics of patients and HCs. Age, sex, and body mass index were comparable among HCs, MMA, and SA (all *p* > 0.05). In contrast, lung function showed a clear severity-dependent decline, with HCs consistently exhibiting the highest values, followed by MMA and SA. This trend was observed for forced vital capacity (FVC)% predicted, forced expiratory volume in 1 s (FEV_1_)% predicted, FEV_1_/FVC, peak expiratory flow (PEF), and all maximal expiratory flow (MEF) indices (all *p* < 0.001). Asthma control worsened with severity, as reflected by lower Asthma Control Test (ACT) scores in SA compared with MMA (*p* < 0.001). Fractional exhaled nitric oxide (FeNO) levels were higher in SA than in MMA (*p* = 0.045). Compared with HCs and patients with MMA, GSHr in the sputum of patients with SA was significantly reduced (Figure 1B).

Correlation analysis exhibited that GSH was negatively associated with FeNO and sputum neutrophils, while positively associated with PEF, FEV_1_/FVC%, and ACT (Figure 1C–F, Table S2). Furthermore, the receiver operating characteristic (ROC) curve exhibited a significant diagnostic potential of GSH for SA (AUC = 0.860, 95% CI: 0.80–0.92, *p* < 0.001) (Figure 1G, Table S3).

To elucidate the role of GSHr in different asthma inflammatory phenotypes, we conducted subgroup analysis based on asthma inflammation phenotype (Figure 1H). Compared with eosinophilic asthma, the sputum GSHr levels of mixed granulocytic asthma were decreased (Figure 1I). Together, these findings identify sputum GSHr as a severity-linked redox marker that reflects airway inflammatory phenotypes and holds potential clinical utility in distinguishing severe asthma.

### 3.2. Identification of GSH-Metabolizing Enzymes as Potential Clinical Biomarkers in SA Using the Unbiased BIOmarkers in Prediction of REspiratory Disease Outcomes Project

**Figure 2:**
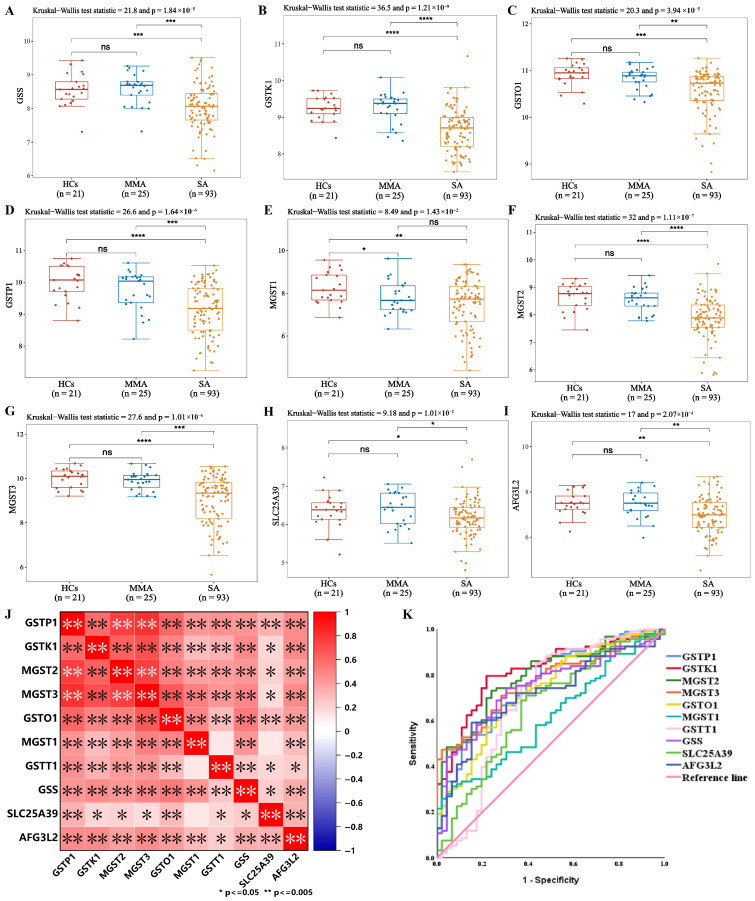
Identification of GSH-metabolizing enzymes as potential clinical biomarkers in SA using the Unbiased BIOmarkers in Prediction of REspiratory Disease Outcomes Project. (**A**–**I**) Expression levels of *GSS*, *GSTK1*, *GSTO1*, *GSTP1*, *MGST1*, *MGST2*, *MGST3*, *AFG3L2*, and *SLC25A39* in the sputum samples of transcriptomics. (**J**) Correlation analysis between GSH-metabolizing enzymes. (**K**) ROC curve for sputum GSH-metabolizing enzymes in predicting the diagnosis of severe asthma. * *p* < 0.05, ** *p* < 0.005, *** *p* < 0.0005, **** *p* < 0.00005, ns > 0.05.

Motivated by the sputum GSHr results, we next interrogated whether the GSH-metabolizing enzymes might underlie the observed redox alterations and serve as candidate clinical biomarkers for SA by RNA sequencing data from the Unbiased BIOmarkers in Prediction of REspiratory Disease Outcomes Project (U-BIOPRED) framework, which was a large and complex research project undertaken to understand severe asthma [29]. As shown in Figure 2A, transcriptomic analysis of induced sputum samples (n = 139) from the U-BIOPRED project (GSE76262) revealed significantly decreased levels of GSH synthetase (GSS), glutathione-S-transferase (GSTK1, GSTO1, GSTP1, MGST1, MGST2, MGST3), mitochondrial GSH transporter (AFG3L2, SLC25A39) in SA compared to HCs and MMA [30,31,32]. The pairwise correlation heatmap demonstrated strong positive correlations among most glutathione-S-transferase (GST) superfamily members, GSS, and mitochondrial GSH transporter, indicating that these enzymes tend to be co-regulated within the GSH metabolic network (Figure 2B). ROC curve analysis further identified GSH-metabolizing enzymes above as potential discriminators of SA, each achieving moderate to high diagnostic performance (Figure 2C, Table S4).

Building on these findings, we sought to assess the robustness of these GSH-metabolizing enzymes as diagnostic features by incorporating them into machine-learning-based classification models. To this end, we applied the CatBoost algorithm, which is well-suited for handling heterogeneous clinical transcriptomic data. By integrating the expression profiles of the identified metabolic enzymes into the CatBoost framework, we found that the CatBoost classification model exhibited perfect performance on the training dataset, with all three sample classes accurately distinguished and no misclassifications observed (Figure S1A). The CatBoost classification model achieved the best performance for the severe asthma class: all 27 true cases were correctly identified, with only one instance misclassified as another category (a false negative), indicating a sensitivity of 96.4% (Figure S1B). The CatBoost model showed clear overfitting. Training set metrics were perfect, while cross-validation accuracy and recall dropped to 0.67, F1 to 0.626, and AUC to 0.41, indicating poor generalizability. On the test set, accuracy and recall were 0.738, precision was 0.722, F1 was 0.693, and AUC was 0.851, reflecting moderate discriminative ability but limited stability, likely due to insufficient separation of mild-to-moderate asthma and healthy controls (Figure S1C,D, Table S2). These findings support the potential of GSH-metabolizing enzymes as candidate biomarkers for severe asthma, with the CatBoost model serving to illustrate their discriminative capacity.

### 3.3. Dysregulation of GSH Metabolism in the TDI-Induced SA Model

**Figure 3:**
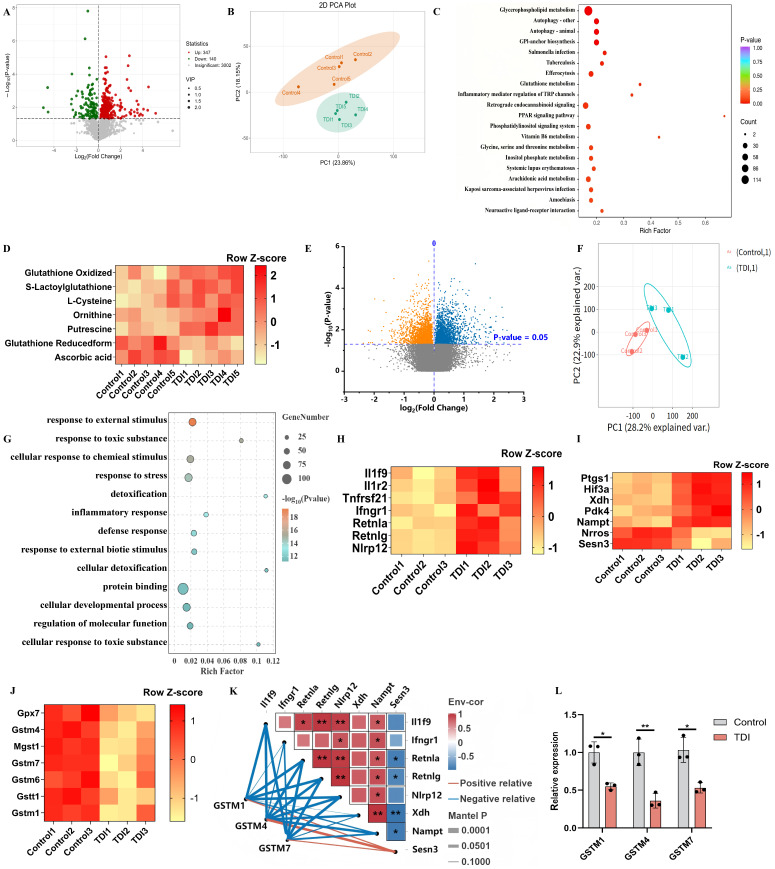
Dysregulation of GSH metabolism in the TDI-induced SA model. (**A**) Volcano analysis of metabolomics (TDI vs. control). (**B**) Principal component analysis (PCA) of metabolomics. (**C**) KEGG pathway enrichment of metabolomics. (**D**) Heatmap analysis of differentially expressed metabolites. (**E**) Volcano analysis of transcriptomics (TDI vs. control). The blue dots represent upregulated genes, and the orange dots represent downregulated genes. (**F**) PCA of transcriptomics. (**G**) GO pathway enrichment of transcriptomics. (**H**–**J**) Heatmap analysis of differentially expressed genes. (**K**) Heat map of correlation analysis between differentially expressed genes. (**L**) mRNA expression levels of GSTM1, GSTM4, GSTM7. A mixture of 2 vol of acetone and 3 vol of olive oil was used as the vehicle (control). * *p* < 0.05, ** *p* < 0.005, ns > 0.05.

To further refine the link between GSH dysregulation and mitochondrial dysfunction, we next performed mitochondrial metabolomic analysis to characterize mitochondrial GSH metabolism in the TDI-induced severe asthma model. Metabolomic analysis of the TDI-induced SA model revealed distinct metabolic features between the TDI-induced SA group and controls, as indicated by volcano plots, principal component analysis (PCA), and hierarchical clustering (Figure 3A,B). Kyoto Encyclopedia of Genes and Genomes (KEGG) pathway enrichment analysis suggested a significant enrichment of the autophagy and GSH metabolism pathway, accompanied by elevated levels of GSH oxidized and decreased levels of GSHr (Figure 3C,D). Transcriptomic analysis of the TDI-induced SA model revealed significant differences in gene expression profiles between the TDI-induced SA group and controls, as indicated by volcano plots and PCA (Figure 3E,F). Gene Ontology (GO) pathway enrichment analysis exhibited a significant enrichment of inflammatory response and cellular response to chemical stimulus (Figure 3G). Heatmap analysis of differentially expressed genes indicated significant activation of mixed granulocytic inflammation-related genes (*Il1f9, Il1r2, Tnfrsf21, Ifngr1, Retnla, Retnlg, Nlrp12*) [33,34,35,36], oxidative stress-related genes (*Ptgs1, Hif3a, Xdh, Nampt, Nrros, Sesn3*) [33,37], along with suppression of GST-related genes (*Gstm4, Mgst1, Gstm7, Gstm1, Gstt1*) in the TDI-induced SA group, compared to the control group (Figure 3H–J). Correlation analysis revealed significant associations between GST-related genes and oxidative stress- or mixed granulocytic inflammation-related genes (Figure 3K). Quantitative real-time PCR (qPCR) further confirmed the suppressive effect of TDI on GST expression (Figure 3L). Together, these results demonstrate that TDI-induced asthma is characterized by disrupted GSH metabolism, GST suppression, and enhanced oxidative stress and mixed granulocytic inflammation.

**Figure 4:**
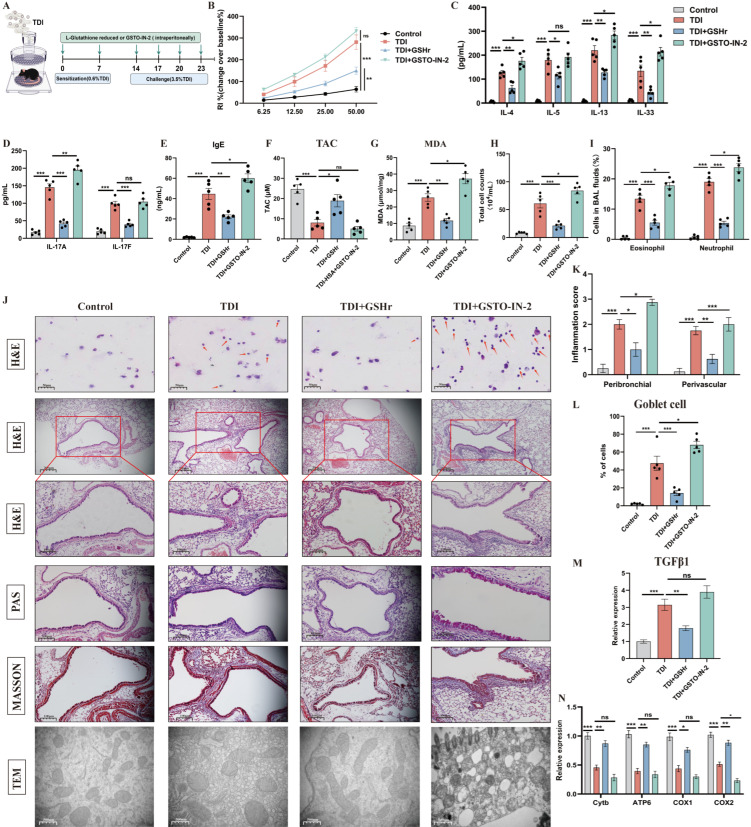
Inhibition of GST aggravates airway inflammation and mitochondrial dysfunction. (**A**) Schematic diagram representation of experimental plan (By Figdraw). (**B**) AHR was measured by lung resistance (RL). Results are shown as a percentage over baseline value (n = 5). (**C**–**G**) Measurement of IgE, IL-4, IL-5, IL-13, IL-33, TAC, MDA, and IL-17 (n = 5). (**H**,**I**) Total and differential inflammatory cells in BALF (n = 5). Percentages of different inflammatory cells were calculated by counting a total of 200 cells in H&E-stained cytospin samples. (**J**) Representative H&E-stained lung sections of different groups, periodic acid–Schiff base (PAS) stained sections, and Masson’s trichrome stained sections. The red arrows indicate eosinophils or neutrophils. Transmission electron microscopy detection of different groups at 40,000×. (**K**,**L**) Semi-quantitative analysis of airway inflammation and goblet cell metaplasia. (**M**) mRNA expression of TGFβ1. (**N**) mRNA expression of mitochondrial DNA-encoded cytochrome b (Cytb), ATP synthase 6 (ATP6), cytochrome c oxidase 1 (COX1), and cytochrome c oxidase 2 (COX2) in the whole lung. A mixture of 2 vol of acetone and 3 vol of olive oil was used as the vehicle (control). * *p* < 0.05, ** *p* < 0.005, *** *p* < 0.0005, ns > 0.05, n = 5–8.

To validate the functional relevance of GST suppression and GSH dysregulation observed in the TDI model, we next assessed the effects of pharmacological modulation of GST (GSTO-IN-2) and GSHr (Figure 4A). TDI-exposed mice showed elevated levels of airway responsiveness, IgE, IL-33, IL-4, IL-5, IL-13, IL-17, malonaldehyde (MDA), inflammatory cells, and decreased levels of total antioxidant capacity (TAC) (Figure 4B–J). Histopathological and semi-quantitative analyses revealed that TDI exposure induced characteristic features of airway inflammation, including peribronchial and perivascular inflammatory cell infiltration, mucus hypersecretion, collagen deposition, and mitochondrial dysfunction, as demonstrated by hematoxylin and eosin (H&E), periodic acid–Schiff (PAS), and Masson’s trichrome staining, respectively (Figure 4J–M). Furthermore, transmission electron microscopy (TEM) and qPCR revealed airway mitochondrial dysfunction of TDI-exposed mice (Figure 4J,N), characterized by mitochondrial cristae disorganization and swelling, and decreased expression of mitochondrial steady-state-related genes. Administration of GSTO-IN-2 markedly aggravated these changes, while GSHr ameliorated them.

**Figure 5:**
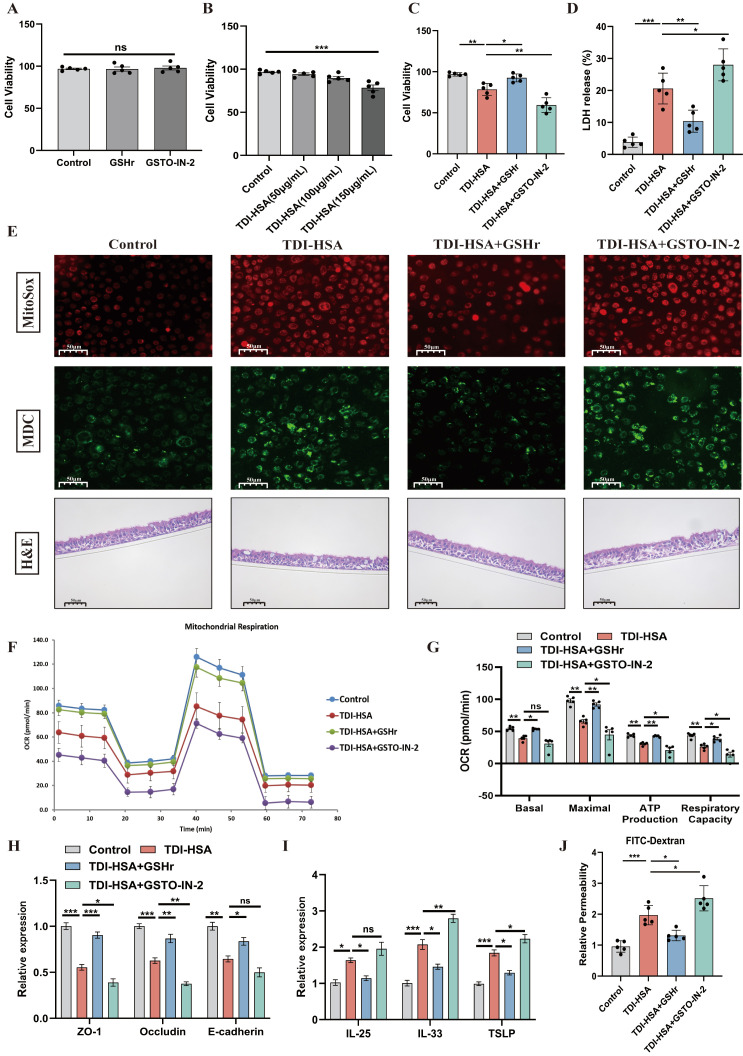
Effect of targeting the GSH metabolism on TDI-HSA-treated HAECs. (**A**–**C**) Detection of cytotoxicity by CCK8 of different groups. (**D**) Measurement of the LDH release rate of different groups. (**E**) MitoSOX and MDC stained HAECs of different groups. Representative H&E-stained HAECs of different groups at 400× original magnification. (**F**,**G**) Analysis of oxygen consumption rate (OCR) by Seahorse assay. (**H**,**I**) qPCR analysis of the mRNA expression of TSLP, IL-25, IL-33, E-cadherin, ZO-1 and occludin of the HAECs. (**J**) Relative permeability of HAECs detected by FITC-dextran. GSTO-IN-2 (20 μM), L-glutathione (1 mM). PBS was used as the vehicle (control). * *p* < 0.05, ** *p* < 0.005, *** *p* < 0.0005, ns > 0.05, n = 5.

In primary human airway epithelial cells (HAECs), TDI–human serum albumin (TDI-HSA) induced cell death, lactate dehydrogenase (LDH) release, and autophagy (with MDC staining) at a concentration of 150 μg/mL (Figure 5A–E). MitoSOX staining also indicated an increase in mitochondrial oxidative production of TDI-HSA-treated HAECs (Figure 5E). Furthermore, TDI-HSA decreased the levels of mitochondrial oxygen consumption and ATP production in HAECs (Figure 5F,G). We then cultured HAECs on an air–liquid interface (ALI) and co-cultured them with TDI-HSA, observing a diminution in barrier-related molecules (ZO-1, occludin, and E-cadherin) and an increase in airway epithelial alarmins (IL-25, IL-33, and TSLP) and cellular permeability (Figure 5F,H–J). Administration of GSTO-IN-2 markedly exacerbated these changes, while GSHr ameliorated them. Collectively, these findings demonstrate that TDI-induced asthma is driven by profound dysregulation of GSH metabolism and GST suppression, which exacerbate oxidative stress, inflammation, mitochondrial dysfunction, and epithelial barrier disruption, while restoration of GSH homeostasis confers protective effects in both in vivo and in vitro models.

### 3.4. TDI Inhibits SLC25A39 to Induce GSH Transport and Mitochondrial Dysfunction

**Figure 6:**
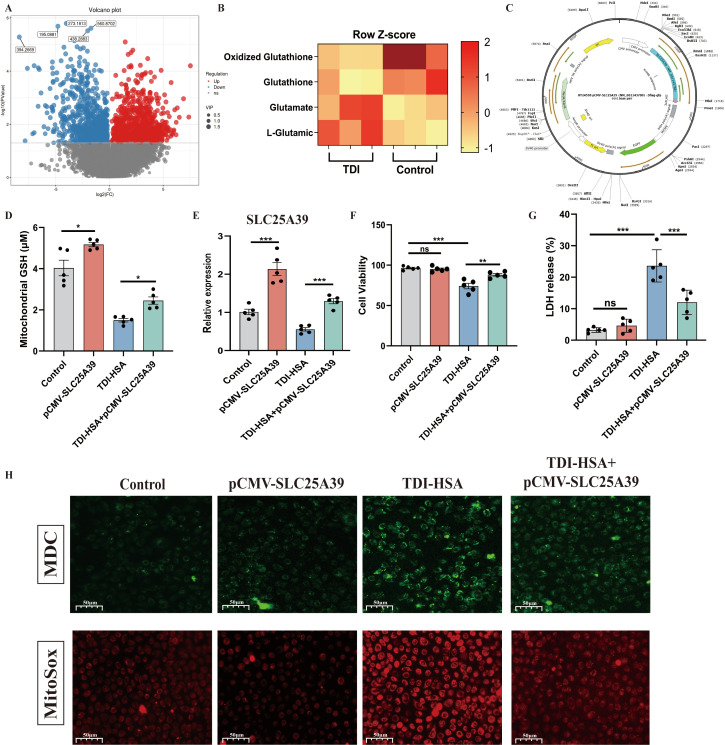
TDI inhibits SLC25A39 to induce GSH transport and mitochondrial dysfunction. (**A**) Volcano plot of untargeted metabolomics of the mitochondria extracted from healthy and TDI-exposed mice. (**B**) Heat map analysis of oxidized glutathione, glutathione, glutamate, and L-glutamic acid of untargeted metabolomics. (**C**) Construction diagram of the SLC25A39 overexpression plasmid. Asterisk (*) indicates a restriction enzyme site that can be recognized and cleaved by both BspDI and ClaI. (**D**) Measurement of mitochondrial GSH. (**E**) qPCR analysis of the mRNA expression of SLC25A39 in the HAECs. (**F**) Measurement of LDH release rate in the HAECs. (**G**) Detection of cytotoxicity by CCK8 of different groups. (**H**) MDC and MitoSox stained HAECs of different groups. PBS was used as the vehicle (control). * *p* < 0.05, ** *p* < 0.005, *** *p* < 0.0005, ns > 0.05.

To further clarify the role of GSH metabolism in oxidative stress in asthma, untargeted metabolomic analysis was performed on mitochondria isolated from the lung tissue of TDI-induced asthmatic mice (Figure 6A). As shown in Figure 6B, mitochondrial total GSH and oxidized GSH were significantly decreased by exposure to TDI. SLC25A39 was necessary for mitochondrial GSH transport to maintain GSH redox cycling and mitochondrial homeostasis [38]. Hence, we established an over-expression plasmid of SLC25A39 to treat HAECs (Figure 6C).

Mitochondrial GSH and SLC25A39 were inhibited by TDI-HSA in HAECs (Figure 6D,E). MDC and MitoSox staining indicated that TDI-HSA induced mitochondrial oxidative production and cellular autophagy (Figure 6H), accompanied by elevated levels of LDH release and cell death (Figure 6F,G). However, over-expression of SLC25A39 significantly inhibited these changes above. The results above indicated that TDI-HSA inhibited SLC25A39 to induce abnormal GSH transport, mitochondrial dysfunction, and cellular autophagy.

### 3.5. Computational Identification and Experimental Validation of Olopatadine and Dabrafenib as Nrp1-Targeting Candidates

Neuropilin-1 (Nrp1) is a key regulator of mitochondrial transport and oxidative stress [15], suggesting a close link to GSH metabolic homeostasis. In line with this notion, airway epithelial transcriptomic profiling (GSE63142) revealed a marked upregulation of NRP1 in patients with severe asthma compared with healthy controls, accompanied by a coordinated downregulation of multiple glutathione S-transferase genes, including GSTA1, GSTA2, GSTA3, GSTA5, and GSTM2. Correlation analyses further demonstrated a significant inverse relationship between NRP1 expression and GST family members, indicating that enhanced NRP1 expression is tightly associated with suppression of GSH-dependent detoxification pathways in the severe asthma airway epithelium. In parallel, ROC curve analyses showed that reduced expression levels of these GST genes could effectively distinguish patients with severe asthma from controls, with all GSTs achieving statistical significance (*p* < 0.05) and area under the curve (AUC) values greater than 0.6 (Table S5, Figure S2). Together, these findings identify a coordinated NRP1 upregulation and GST downregulation signature in severe asthma, linking NRP1 to disrupted GSH metabolism. This provides the rationale for subsequent validation of NRP1-dependent regulation of mitochondrial function and GSH metabolism in experimental models of severe asthma.

Considering that there were few Nrp1 b1 domain-targeting inhibitors suitable for the clinic, we tried to identify clinical drugs that target the b1 domain of Nrp1. We found that the Asp320, Thr349, Tyr353, Ser346, Lys351, and Ile146 residues were located in the active region in the b1 domain of Nrp1, which were selected for the targets of the inhibitors of the b1 domain. The threshold autodock score was between −9.00 and −7.80, and the threshold libDock score was over 100. Seven compounds that screened met the docking scores were identified as potential ligands for the b1 domain of Nrp1 (Figure S3A, Table S6). Olopatadine and dabrafenib were selected for further verification.

To further clarify the binding stability between the screened drugs and targets, we performed root-mean-square deviation (RMSD) analysis by GROMACS-based molecular dynamics (MD) simulations. The results revealed minimal atomic fluctuations with RMSD values stabilizing within 0.2 nm, indicating stable binding modes (Figure S3B). Nrp1–ligand interactions are shown in Figure S1C and Supplemental Videos S1 and S2, which specifically depict the trajectory of the last 10 ns of the molecular dynamics simulation. The observed fluctuations in RMSD were further supported by gmx_MMPBSA analysis (Table S7). Analysis of gmx_MMPBSA showed enhanced binding affinities of Nrp1–ligand interactions, as evidenced by converged RMSD values and low binding free energy among the tested compounds. Furthermore, cellular thermal shift assays (CETSA) performed in primary human airway epithelial cells revealed that treatment with olopatadine or dabrafenib significantly increased the thermal stability of NRP1, as evidenced by a marked elevation in its thermal denaturation temperature, indicating direct target engagement (Figure S4A). Consistently, co-immunoprecipitation analyses showed that both compounds markedly inhibited the expression of Nrp1 and attenuated the interaction between Nrp1 and its b1-domain ligand VEGFA in murine lung tissues from both control and TDI-exposed groups (Figure S4B). Hence, we prioritized olopatadine and dabrafenib as highly promising candidates for targeting the b1 domain of Nrp1 to further assess its effect on asthma.

### 3.6. Blockade of Nrp1’s b1 Domain Reverses GSH Metabolism Disorder in the TDI-Induced Asthma Model

Detection of GSHr, GST, and GSS exhibited that TDI exposure led to a decrease in GSH metabolism, and that this was reversed by treatment with olopatadine, dabrafenib, or the inhibitor of Nrp1’s b1 domain (EG00229) (Figure S5A–C). Treatment with olopatadine, dabrafenib, or EG00229 also significantly reversed the decreased levels of SLC25A39 and mitochondrial GSH induced by TDI (Figure S5D–F). Together, these findings show that TDI induces a coordinated suppression of glutathione metabolism and mitochondrial GSH availability, which is accompanied by downregulation of SLC25A39 and can be reversed by targeting the Nrp1 b1 domain.

### 3.7. Blockade of Nrp1’s b1 Domain Reduces Airway Inflammation and Mitochondrial Dysfunction in the TDI-Induced Asthma Model

**Figure 7:**
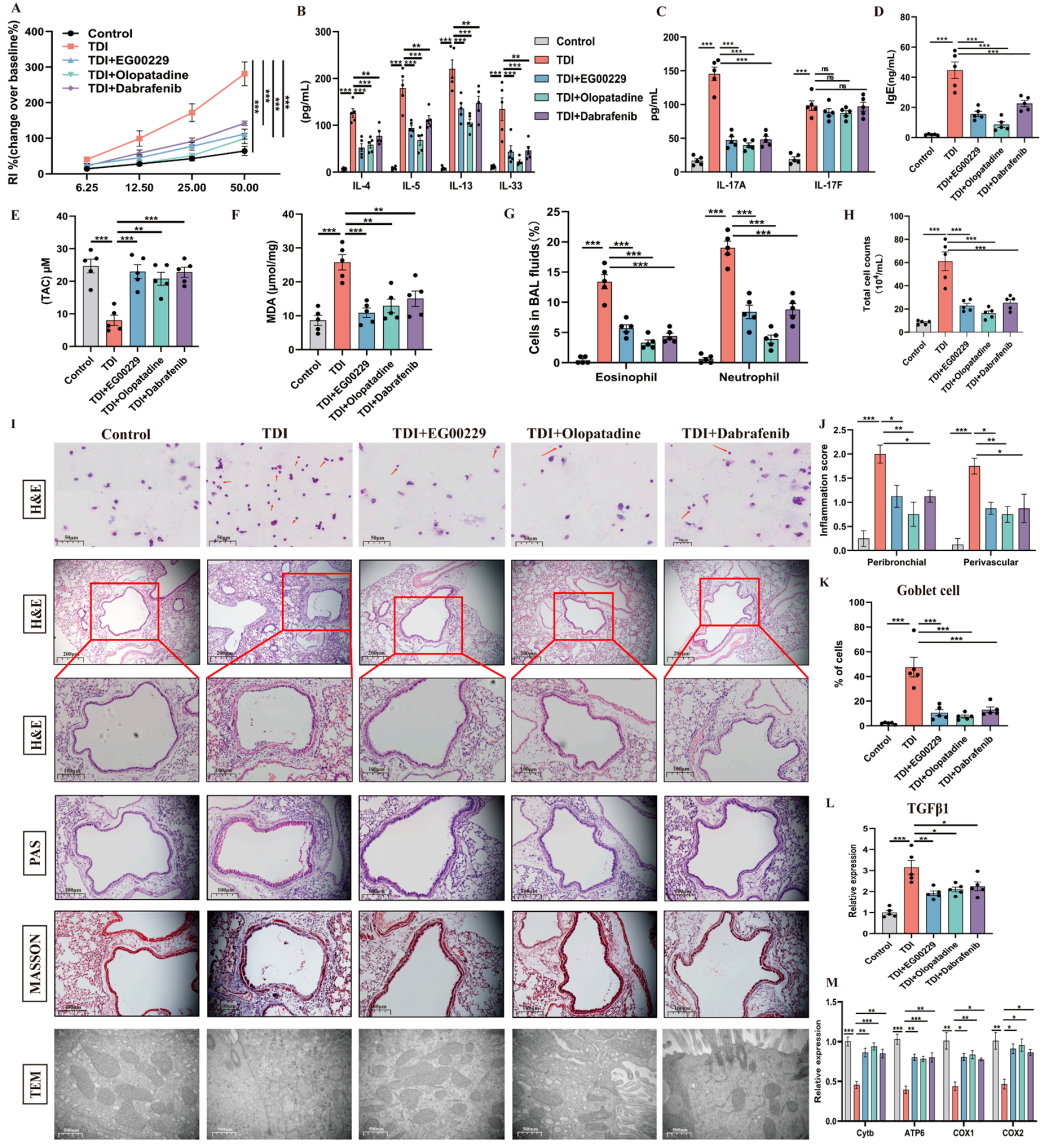
Blockade of the b1 domain of Nrp1 reduces TDI-induced airway inflammation and mitochondrial dysfunction. (**A**) AHR was measured by RL. Results are shown as a percentage over baseline value (n = 5). (**B**–**F**) Measurement of IgE, IL-4, IL-5, IL-13, IL-33, TAC, MDA, and IL-17 (n = 5). (**G**,**H**) Total and differential inflammatory cells in BALF (n = 5). Percentages of different inflammatory cells were calculated by counting a total of 200 cells in H&E-stained cytospin samples. (**I**) Representative H&E-stained lung sections of different groups, PAS-stained sections, and Masson’s trichrome-stained sections. The red arrows indicate eosinophils or neutrophils. Transmission electron microscopy detection of different groups at 40,000×. (**J**,**K**) Semi-quantitative analysis of airway inflammation and goblet cell metaplasia. (**L**) mRNA expression of TGFβ1. (**M**) mRNA expression of mitochondrial Cytb, ATP6, COX1, and COX2 in the whole lung. A mixture of 2 vol of acetone and 3 vol of olive oil was used as the vehicle (control). * *p* < 0.05, ** *p* < 0.005, *** *p* < 0.0005, ns > 0.05, n = 5–8.

As shown in Figure 7A–I, our data portrayed pharmacological inhibition of Nrp1’s b1 domain with EG00229, olopatadine, or dabrafenib markedly reversed TDI-induced elevated levels of airway responsiveness, IgE, IL-33, IL-4, IL-5, IL-13, IL-17, MDA, inflammatory cells, and decreased levels of TAC. Furthermore, histopathological and semi-quantitative analyses revealed that treatment with EG00229, olopatadine, or dabrafenib substantially attenuated TDI-induced airway inflammation, airway remodeling, goblet cell metaplasia, and mitochondrial dysfunction (Figure 7I–M).

To further delineate the domain-specific contribution of Nrp1 to TDI-induced airway pathology, we next compared the effects of pharmacological inhibitors targeting distinct Nrp1 domains. Treatment with EG01377 dihydrochloride, an inhibitor of Nrp1’s a1 and b1 domains, could not alleviate TDI-induced airway inflammation and AHR (Figure S6). Together, these findings indicate that selective inhibition of the Nrp1 b1 domain, rather than broad domain blockade, is critical for mitigating TDI-induced airway inflammation, remodeling, oxidative stress, and mitochondrial dysfunction.

**Figure 8:**
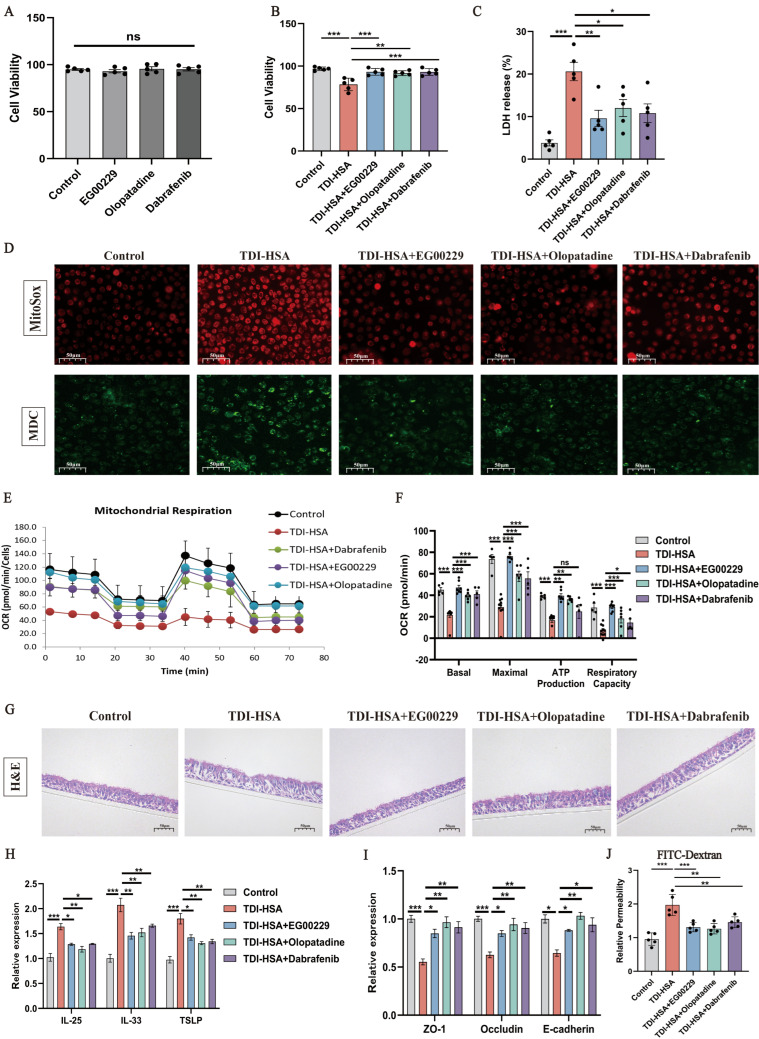
Effects of targeting the b1 domain of Nrp1 on TDI-HSA-treated primary human airway epithelial cells. (**A**,**B**) Detection of cytotoxicity by CCK8 of different groups. (**C**) Measurement of the LDH release rate of different groups. (**D**) MitoSOX and MDC stained primary human airway epithelial cells (HAECs) of different groups. (**E**,**F**) Analysis of OCR by Seahorse assay. (**G**) Representative H&E-stained HAECs of different groups at 400× original magnification. (**H**,**I**) qPCR analysis of the mRNA expression of TSLP, IL-25, IL-33, E-cadherin, ZO-1 and occludin of the HAECs. (**J**) Relative permeability of HAECs detected by FITC-dextran. EG00229 (5 μM), olopatadine (0.1 mM), dabrafenib (0.5 μM). PBS was used as the vehicle (control). * *p* < 0.05, ** *p* < 0.005, *** *p* < 0.0005, ns > 0.05.

In TDI-HSA-treated monolayer HAECs, treatment with EG00229, olopatadine, or dabrafenib markedly ameliorated cell deaths, LDH release, autophagy, and mitochondrial oxidative stress (Figure 8A–D). TDI-HSA also diminished the levels of oxygen consumption and ATP production in HAECs (Figure 8E,F). In TDI-HSA-treated ALI-cultured HAECs, the airway epithelial barrier was significantly disrupted, and this was accompanied by a drop in epithelial barrier-molecule expression and a rise in alarm-protein expression and cellular permeability (Figure 8G–J). However, the aforementioned changes were largely circumvented by pretreatment with EG00229, olopatadine, or dabrafenib. Collectively, these results demonstrate that pharmacological inhibition of the Nrp1’s b1 domain protects airway epithelial cells from TDI-induced mitochondrial dysfunction, oxidative stress, autophagy, and barrier disruption, thereby reinforcing its critical role in epithelial injury responses.

### 3.8. Dysregulation of GSH Metabolism in the HDM/LPS-Induced SA Model

To validate our findings beyond the TDI-induced severe asthma model, we further examined GSH metabolism in an HDM/LPS-induced SA model. Metabolomic analysis of the HDM/LPS-induced SA model revealed distinct metabolic features between the HDM/LPS-induced SA group and controls, as indicated by volcano plots and PCA (Figure S7A,B). Heatmap analysis of differentially expressed metabolites exhibited elevated levels of oxidized GSH and decreased levels of GSHr in the HDM/LPS-induced SA group compared to the control group (Figure S7C). Transcriptomic analysis of the HDM/LPS-induced SA model revealed significant differences in gene expression profiles between the TDI asthma group and controls, as indicated by volcano plots and PCA (Figure S7D,E). Heatmap analysis of differentially expressed genes indicated significant activation of mixed granulocytic inflammation-related genes (*Il1b, Il6, Il17a, Ifngr2, Il1a, Il33, Il13*), oxidative stress-related genes (*Nfkb1, Homx1, Gclc, Akr1b8, Ptgr1, Nqo1*) along with suppression of GST-related genes (*Gstm1, Gstm2, Gstm6, Gstt1, Gsta2, Gsta3*) in the HDM/LPS-induced SA group, compared to the control group (Figure S7F–H). Correlation analysis revealed significant associations between GST-related genes and oxidative stress– or mixed granulocytic inflammation–related genes (Figure S7I). Together, these data confirm that dysregulated GSH metabolism and pronounced redox imbalance, characterized by GSH depletion, GST suppression, and heightened oxidative stress and mixed granulocytic inflammation, are a conserved feature of severe asthma across distinct experimental models.

**Figure 9:**
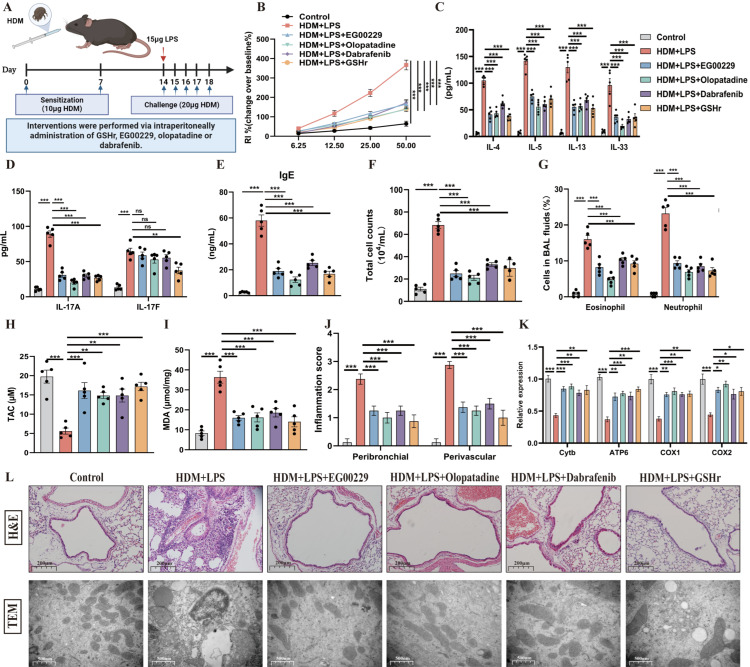
Targeting the Nrp1-GSH axis reduces HDM/LPS-induced airway inflammation. (**A**) Schematic diagram representation of experimental plan (by Biorender). (**B**) AHR was measured by RL. Results are shown as a percentage over baseline value (n = 5). (**C**–**E**) Measurement of IgE, IL-4, IL-5, IL-13, IL-33, and IL-17 (n = 5). (**F**,**G**) Total and differential inflammatory cells in BALF (n = 5). (**H**,**I**) Measurement of MDA and TAC by ELISA (n = 5). (**J**) Semi-quantitative analysis of airway inflammation. (**K**) mRNA expression of mitochondrial Cytb, ATP6, COX1 and COX2 in the whole lung. (**L**) Representative H&E-stained lung sections of different groups at 100× original magnification. Transmission electron microscopy detection of different groups at 40,000×. PBS was used as the vehicle (control). * *p* < 0.05, ** *p* < 0.005, *** *p* < 0.0005, ns > 0.05.

To elucidate the potential of targeted Nrp1-GSH therapy for severe asthma, we administered EG00229, olopatadine, dabrafenib, and GSHr on HDM/LPS-induced SA model (Figure 9A). Consistent with the TDI-induced SA model, the HDM-LPS-exposed mice exhibited elevated levels of airway responsiveness, IgE, IL-33, IL-4, IL-5, IL-13, IL-17, MDA, inflammatory cells, and decreased levels of TAC (Figure 9B–I). Furthermore, histopathological and semi-quantitative analyses revealed that HDM/LPS exposure induced characteristic features of airway inflammation and mitochondrial damage (Figure 9J–L). Collectively, these findings indicate that pharmacological restoration of Nrp1-GSH signaling effectively mitigates redox imbalance, inflammation, and mitochondrial injury in the HDM/LPS-induced severe asthma model, corroborating its therapeutic relevance across disease contexts.

### 3.9. The Mechanism of the β-Catenin-Nrp1 Axis in Regulating the TDI-Induced Asthma Model

Previous studies have demonstrated that TGFβ1 and VEGF activate Nrp1 through binding to its b1 domain [13], with TGFβ1 additionally inducing the expression of MMP2 and MMP9 [39]. Notably, these molecules were identified as β-catenin downstream targets in our prior investigation [2], suggesting the potential interaction between β-catenin and Nrp1. As shown in Figure S8A, pharmacological inhibition of β-catenin using ICG-001 significantly inhibited the elevated expression of Nrp1 and its b1 domain ligands (VEGF and TGFβ1) in TDI-induced asthmatic mice. Furthermore, ICG-001 largely reversed the decreased level of rGSH, GST, GSS, and SLC25A39 induced by TDI (Figure S8B–D). Hence, β-catenin might activate Nrp1 to inhibit GSH metabolism by targeting its b1 domain in the TDI asthma model.

To further delineate the regulatory hierarchy, we examined the effects of downstream pathway inhibitors. Administered with EG00229, olopatadine, or dabrafenib significantly reduced TDI-induced upregulation of TGFβ1, VEGF, MMP2, and MMP9 (Figure S8E). STRING (https://string-db.org/) protein–protein interaction network pathway analysis prompted β-catenin to regulate Nrp1 through MMP2, MMP9, VEGFA, and TGFβ1 (Figure S8F). Taken together, the evidence above supported a mechanistic cascade wherein β-catenin exerted its regulatory effects on Nrp1 through these targets.

## 4. Discussion

Severe asthma is a clinically heterogeneous disease in which persistent inflammation, oxidative stress, and mitochondrial dysfunction contribute to disease refractoriness. In this study, by integrating clinical profiling, multi-omics analyses, experimental models, and computational drug screening, we demonstrate that dysregulated GSH metabolism represents a central and conserved pathogenic mechanism in severe asthma, mechanistically linking redox imbalance, mitochondrial dysfunction, epithelial barrier disruption, and airway inflammation.

Our clinical findings identify sputum GSHr as a severity-associated redox marker in asthma. GSHr levels were significantly decreased in patients with severe asthma compared with HCs and patients with MMA, and correlated positively with lung function indices and asthma control, while correlating negatively with FeNO and sputum neutrophils. These associations suggest that GSH depletion reflects not only oxidative stress burden but also disease activity and functional impairment. Notably, GSHr levels were lowest in mixed granulocytic asthma compared with eosinophilic asthma, indicating that redox imbalance may be particularly relevant in non-type 2 inflammatory phenotypes that are often refractory to corticosteroids. The strong diagnostic performance of sputum GSHr in ROC analysis further supports its potential clinical utility as a biomarker for identifying severe, redox-driven asthma. Compared with previous studies [40], our findings are consistent with earlier reports, demonstrating that the levels of GSHr in patients with mild-to-moderate asthma are comparable to those in healthy controls. In contrast, severe asthma is characterized by glutathione depletion, manifested as a marked imbalance between GSHr and oxidized glutathione. Building upon these observations, we further elucidated the associations between GSH status and relevant clinical characteristics as well as inflammatory phenotypes.

Building upon these observations, transcriptomic analysis of induced sputum samples from the U-BIOPRED cohort revealed coordinated downregulation of multiple GSH-metabolizing enzymes, including GSS, several GST isoforms, and mitochondrial GSH transport–related genes. The strong positive correlations among these enzymes indicate a systemic collapse of the GSH metabolic network rather than isolated enzymatic defects. Machine-learning–based classification further demonstrated that these GSH-related genes possess discriminative capacity for severe asthma, although limited generalizability likely reflects phenotypic overlap between mild asthma and healthy controls. Collectively, these findings position impaired GSH metabolism as a molecular hallmark of severe asthma.

Importantly, our study moves beyond the correlative observation of GSH depletion as a redox imbalance and provides converging evidence supporting its role as a functional determinant of disease severity. While clinical data show that reduced sputum GSH is closely associated with airflow limitation, poor asthma control, and increased inflammatory burden, such associations alone are insufficient to establish causality. To address this, we combined multi-omics profiling with targeted functional perturbations to delineate the causal role of GSH metabolism.

At the systems level, integrated mitochondrial metabolomic and transcriptomic analyses revealed a coordinated disruption of the GSH metabolic network, characterized by reduced GSHr, accumulation of oxidized GSH, suppression of GST expression, and activation of oxidative stress and mixed granulocytic inflammatory pathways. This concerted dysregulation suggests that GSH depletion reflects an active metabolic reprogramming rather than a passive consequence of oxidative stress.

Critically, causal evidence is provided by bidirectional pharmacological interventions. Inhibition of GST further exacerbated airway hyperresponsiveness, mitochondrial damage, oxidative stress, and epithelial barrier disruption, whereas GSHr supplementation markedly ameliorated these pathological features in both in vivo and in vitro models. Together with prior studies demonstrating the anti-inflammatory and therapeutic effects of GSH supplementation in airway diseases, these findings indicate that modulation of GSH metabolism is sufficient to drive or reverse key disease phenotypes. Therefore, GSH metabolism functions not merely as a biomarker but as an active regulatory node controlling disease progression.

Mechanistically, our data further establish a direct link between GSH metabolism and mitochondrial integrity, which underlies its impact on inflammatory amplification. Leveraging untargeted mitochondrial metabolomic profiling, we observed a marked depletion of mitochondrial GSH following TDI exposure despite adequate cytosolic substrate availability, arguing against a simple feedback-driven consumption model. Instead, this uncoupling points to impaired mitochondrial GSH transport. Consistently, SLC25A39, an essential mitochondrial GSH transporter, was significantly suppressed in both in vivo and in vitro models, leading to mitochondrial GSH deficiency, excessive mitochondrial ROS production, autophagy activation, and epithelial injury. Notably, enforced overexpression of SLC25A39 restored mitochondrial GSH levels and reversed mitochondrial dysfunction and cellular damage, providing direct rescue evidence for causality.

Functionally, mitochondrial GSH depletion-driven ROS overproduction serves as a key upstream trigger for inflammatory amplification, promoting epithelial alarmin release and mixed granulocytic inflammation, thereby establishing a feed-forward loop that exacerbates airway pathology. Taken together, these findings support a hierarchical model in which dysregulated GSH metabolism actively governs mitochondrial homeostasis and downstream inflammatory amplification. Thus, GSH metabolism is not merely a passive indicator of oxidative stress but a central pathogenic driver that directly regulates mitochondrial integrity and disease severity in severe asthma.

In addition to intrinsic defects in glutathione metabolism, our data identify Nrp1 as an upstream regulatory hub linking redox imbalance to mitochondrial dysfunction and airway inflammation in severe asthma. We exhibited that TDI-induced asthma suppressed mitochondrial GSH transport and GST expression through the b1 domain of Nrp1. Selective pharmacological inhibition of the Nrp1’s b1 domain using EG00229, olopatadine, or dabrafenib restored GSH metabolism, mitochondrial function, epithelial barrier integrity, and attenuated airway inflammation and remodeling. In contrast, inhibition of both the a1 and b1 domains failed to confer protection, underscoring the importance of domain-specific Nrp1 signaling. The identification of clinically approved drugs as effective Nrp1’s b1 domain-targeting agents underscores their translational potential, offering a low-cost, fast, and readily implementable therapeutic strategy for severe asthma.

Asthma is a highly heterogeneous disease, and SA encompasses multiple distinct inflammatory phenotypes. Although recent advances in type 2 biologics have substantially improved the management of eosinophilic SA, effective targeted therapies remain unavailable for the more complex mixed-granulocytic and neutrophilic subtypes [41]. According to the Global Initiative for Asthma guidelines and the U-BIOPRED consensus [42,43], the diagnosis of SA is primarily defined by AHR, symptom control, and treatment response rather than by the underlying inflammatory profile.

To account for this heterogeneity, we employed two steroid-insensitive murine models with differing inflammatory characteristics and triggers for validation. These models represent complementary etiologies of SA: the TDI model simulates chemically induced, mixed-granulocytic, steroid-insensitive asthma [4,44], whereas the HDM/LPS model recapitulates allergen- and endotoxin-driven mixed-granulocytic inflammation [45]. Notably, the pathological features of GSH depletion, GST suppression, redox imbalance, and mixed granulocytic inflammation were conserved in the HDM/LPS-induced severe asthma model, indicating that these mechanisms are not restricted to chemical-induced asthma. Pharmacological restoration of Nrp1–GSH signaling similarly mitigated airway inflammation, oxidative stress, and mitochondrial injury in this model, supporting the robustness and generalizability of the proposed mechanism across distinct asthma contexts.

We have previously found that β-catenin contributed to the pathophysiology of TDI-induced asthma, accompanied by elevated levels of its target genes (MMP2, MMP9, TGFβ, and VEGF) [2,3]. In this study, our data place β-catenin upstream of Nrp1 in regulating GSH metabolism. Inhibition of β-catenin suppressed Nrp1 expression, restored GSH metabolic enzymes and mitochondrial GSH transport, and reduced downstream mediators such as VEGF, TGFβ1, MMP2, and MMP9. These findings suggest a β-catenin–Nrp1 signaling axis that integrates airway remodeling, oxidative stress, mitochondrial dysfunction, and epithelial injury in severe asthma.

From a clinical perspective, sputum GSHr may serve as a non-invasive biomarker for identifying a redox-driven endotype of severe asthma, particularly in patients with mixed granulocytic or neutrophilic inflammation. It may also enable dynamic monitoring of disease activity and treatment response. In addition, targeting the Nrp1–GSH axis, either through GSH supplementation or pharmacological inhibition of Nrp1, represents a potential therapeutic strategy, with clinically approved drugs offering opportunities for rapid translation via repurposing. Beyond asthma, these findings may have broader relevance to other chronic respiratory diseases characterized by oxidative stress and mitochondrial dysfunction, such as COPD, asthma–COPD overlap, and bronchiectasis. This suggests that dysregulated GSH metabolism may represent a conserved pathogenic mechanism and a potential target for precision therapy across chronic airway diseases.

Despite these findings, several limitations should be noted. The clinical cohort was relatively small and from a single center, which may limit generalizability. Furthermore, leukocyte quantity and phenotype both drive redox imbalance in asthma. While neutrophils cause profound ROS production and GSH depletion, eosinophils also contribute to oxidative stress and GSH depletion in asthma [46]. Thus, interpreting antioxidant capacity requires considering both the extent and composition of inflammation. Future research should quantify how specific cell subsets impact GSH homeostasis across asthma endotypes. Another limitation of this study is that the TDI–HSA-stimulated HAECs model does not fully recapitulate the multicellular complexity of neutrophilic asthma, particularly the interactions between epithelial cells and infiltrating neutrophils. As a reductionist system, it primarily reflects epithelial-intrinsic responses to TDI exposure. Future studies using co-culture systems or more physiologically relevant models are warranted to better capture the in vivo inflammatory microenvironment. In addition, the specificity and long-term safety of Nrp1-targeting strategies require further validation before clinical application.

In summary, this study identifies GSH metabolic failure as a central pathogenic mechanism in severe asthma, mechanistically linking redox imbalance to mitochondrial dysfunction, epithelial barrier disruption, and mixed granulocytic inflammation. Our findings define a coordinated GSH-centered network that integrates detoxification, mitochondrial transport, and compartmentalized redox regulation, whose disruption drives oxidative stress and airway pathology, with Nrp1-dependent inhibition of mitochondrial GSH import as a critical upstream node. Targeting the Nrp1 b1 domain effectively restores this network and alleviates disease severity across experimental models, highlighting the therapeutic potential of the Nrp1–GSH axis in redox-driven, treatment-refractory asthma.

## Figures and Tables

**Table 1:** Characteristics of healthy subjects and patients with asthma. BMI, body mass index; FEV_1_, forced expiratory volume in 1 s; FVC, forced vital capacity; MEF, maximal expiratory flow; PEF, peak expiratory flow; BDT, bronchodilation test; ACT, the Asthma Control Test; FeNO, fractional exhaled nitric oxide. Data are reported in mean ± SD or median (interquartile range). N.A., not applicable. Chi-square test for gender, and Kruskal–Wallis test for all other variables.

	Healthy Controls (HCs)	Mild to Moderate Asthma (MMA)	Severe Asthma (SA)	Overall Test *p*-Value	Significant Paired Comparison Results (*p* ≤ 0.05) ^&^
N	30	45	46		
Age, years	47.4 ± 12.6	48.56 ± 12.2	51.0 ± 16.3	*p* = 0.448	
Gender, Male/Female	17/13	18/27	22/24	*p* = 0.364	
BMI, kg/m^2^	23.0 (3.1)	23.8 (5.6)	23.7 (5.3)	*p* = 0.747	
FVC/predicted, % ^&^	109.9 ± 10.4	96.5 ± 22.7	81.0 ± 17.1	*p* < 0.001	HCs > MMA > SA
FEV_1_/predicted, % ^&^	101.1 (17.2)	81.5 (39.0)	57.5 (25.8)	*p* < 0.001	HCs > MMA, SA
FEV_1_/FVC, % ^&^	94.5 ± 7.2	73.4 ± 13.6	62.5 ± 13.0	*p* < 0.001	HCs > MMA > SA
PEF, % ^&^	101.8 ± 16.6	64.1 ± 19.6	34.5 ± 22.3	*p* < 0.001	HCs > MMA > SA
MEF75/25, % ^&^	75.2 (22.0)	33.5 (37.3)	26.5 (21.4)	*p* < 0.001	HCs > MMA, SA
MEF75, % ^&^	87.6 (30.8)	35.6 (39.3)	30.3 (23.8)	*p* < 0.001	HCs > MMA, SA
MEF50, % ^&^	82.7 (23.3)	34.8 (32.7)	24.2 (22.7)	*p* < 0.001	HCs > MMA, SA
MEF25, % ^&^	65.4 (26.7)	27.4 (26.5)	20.5 (14.6)	*p* < 0.001	HCs > MMA, SA
ACT score ^&^	N.A.	20.0 (4.0)	14.0 (4.0)	*p* < 0.001	MMA > SA
FeNO, ppb	N.A.	38.0 (61.5)	58.5 (83.5)	*p* = 0.045	SA > MMA
BDT, positive/negative/N.A.	N.A.	23/15/7	35/9/2		
Sputum cell count, %					
Neutrophils, % ^&^	33.0 (41.5)	49.0 (40.0)	67.0 (21.5)	*p* < 0.001	SA > MMA > HCs
Eosinophils, % ^&^	0.0 (1.0)	4.0 (15.0)	7.5 (14.5)	*p* < 0.001	MMA, SA > HCs
Macrophages, % ^&^	65.0 (45.3)	38.0 (33.5)	20.0 (18.3)	*p* < 0.001	HCs > MMA > SA
Leukomonocyte, % ^&^	0.0 (0.0)	0.0 (0.0)	0.0 (1.0)	*p* < 0.001	SA > HCs, MMA

^&^ Significant paired comparisons are indicated for results with *p* ≤ 0.05.

## Data Availability

The data that support the findings of this study are available from the corresponding author upon reasonable request. Some data may not be made available because of privacy or ethical restrictions. Transcriptomic data are stored in the BioStudies database (S-BSST1232, https://www.ebi.ac.uk/biostudies/studies/S-BSST1232, accessed on 27 February 2026).

## Supplementary Materials

The following supporting information can be downloaded at: https://www.mdpi.com/article/10.3390/antiox15040463/s1, Figure S1. CatBoost model of GSH-metabolizing enzymes for diagnosis; Figure S2. Human airway epithelial biopsy transcriptomic analysis; Figure S3. Ligands binding the b1 domain of Nrp1; Figure S4. Olopatadine and dabrafenib inhibited the protein binding levels of Nrp1 and VEGFA; Figure S5. Nrp1 regulated GSH metabolism in the TDI-induced asthma model; Figure S6. Effect of EG01377 dihydrochloride on TDI-induced airway inflammation; Figure S7. Dysregulation of GSH metabolism in the HDM/LPS-induced SA model; Figure S8. The mechanism of the β-catenin-Nrp1 axis in regulating TDI-induced asthma model; Table S1. Nucleotide sequences of the primers used for PCR; Table S2. Correlation analysis between sputum GSHr and clinical features; Table S3. Predictive values of sputum GSH for predicting severe asthmatic patients; Table S4. Predictive values of differential genes for predicting severe asthma patients; Table S5. Predictive values of differential genes for predicting severe asthma patients; Table S6. The docking score and predicted protein–ligand interaction of the top eight compounds selected in virtual screening; Table S7. Binding free energies predicted using the MMPBSA approach for Nrp1’s b1 domain complexed with different compounds; Supplemental methods; Regents; Video S1 (olopatadine 80–100 ns); Video S2 (dabrafenib 80–100 ns).

## Abbreviations

The following abbreviations are used in this manuscript:

ACT	Asthma Control Test
AHR	Airway hyperresponsiveness
ALI	Air–liquid interface
ANOVA	Analysis of variance
ATP	Adenosine triphosphate
AUC	Area under the curve
BAL	Bronchoalveolar lavage
CatBoost	Categorical boosting algorithm
CETSA	Cellular thermal shift assay
CI	Confidence interval
FeNO	Fractional exhaled nitric oxide
FEV_1_	Forced expiratory volume in 1 second
FVC	Forced vital capacity
GINA	Global Initiative for Asthma
GO	Gene Ontology
GROMACS	GROningen MAchine for Chemical Simulations
GSH	Glutathione
GSHr	Glutathione reduced form
GSS	Glutathione synthetase
GST	Glutathione S-transferase
HAECs	Human airway epithelial cells
HCs	Healthy controls
HDM	House dust mite
H&E	Hematoxylin and eosin
HSA	Human serum albumin
IL	Interleukin
i.n.	Intranasal
i.p.	Intraperitoneal
KEGG	Kyoto Encyclopedia of Genes and Genomes
LDH	Lactate dehydrogenase
LPS	Lipopolysaccharide
MDA	Malonaldehyde
MD	Molecular dynamics
MEF	Maximal expiratory flow
MMA	Mild-to-moderate asthma
MMPBSA	Molecular mechanics Poisson–Boltzmann surface area
Nrp1	Neuropilin-1
OPLS-DA	Orthogonal partial least squares discriminant analysis
PAS	Periodic acid–Schiff
PCA	Principal component analysis
PEF	Peak expiratory flow
RL	Lung resistance
RMSD	Root-mean-square deviation
ROC	Receiver operating characteristic
SA	Severe asthma
SLC25A39	Solute carrier family 25, member 39
SPF	Specific pathogen-free
TAC	Total antioxidant capacity
TEM	Transmission electron microscopy
TDI	Toluene diisocyanate
TGFβ	Transforming growth factor beta
TSLP	Thymic stromal lymphopoietin
U-BIOPRED	Unbiased BIOmarkers in Prediction of REspiratory Disease Outcomes
