# Supplementary material for: Nrp1 Signaling Reprograms Glutathione Metabolism to Drive Mitochondrial Dysfunction in Severe Asthma
Source: Antioxidants (Basel). 2026 Apr 8;15(4):463. doi: 10.3390/antiox15040463 (PMC13114205; doi:10.3390/antiox15040463)
Supplement: Supplementary file 1 [file antioxidants-15-00463-s001.zip › Reagents.pdf]

Reagents and Tools Table

Reagent/Resource	Reference or Source	Identifier or Catalog Number
C57BL/6J mouse	Zhiyuanbio (China)	CAS #W41331
Primary human airway epithelial cells	ATCC (USA)	CAS #PCS-300-010
anti-VEGFA	proteintech (USA)	Catalog #19003-1-AP
anti-Nrp1	proteintech (USA)	Catalog #84429-5-RR
anti-SLC25A39	ABclonal Technology (China)	Catalog #A15450
anti-β-Tubulin	ABclonal Technology (China)	Catalog #A12289
anti-Nrp1	ABclonal Technology (China)	Catalog #A16697
p-CMV-SLC25A39	Hanyibio (China)	Catalog #HY24598
ICG-001	MedchemExpress (USA)	CAS #780757-88-2
L-Glutathione	MedchemExpress (USA)	CAS #70-18-8
EG01377 dihydrochloride	MedchemExpress (USA)	CAS #2749438-61-5
EG00229	MedchemExpress (USA)	CAS #1018927-63-3
olopatadine	MedchemExpress (USA)	CAS #113806-05-6
dabrafenib	MedchemExpress (USA)	CAS #1195765-45-7
methacholine	MedchemExpress (USA)	CAS #62-51-1
Lipopolysaccharides	MedchemExpress (USA)	Catalog #HY-D1056
PierceTM Classic Magnetic IP/CO-IP Kit	Thermo Scientific (USA)	Catalog #88804
IgE	Thermo Scientific (USA)	Catalog #EMIGHE
IL-33	Multisciences (China)	SKU: EK233HS
IL-4	Multisciences (China)	SKU: EK204HS
IL-5	Multisciences (China)	SKU: EK205HS
IL-17A	Multisciences (China)	SKU: EK217HS
IL-17F	Multisciences (China)	SKU: EK2196
IL-13	Multisciences (China)	SKU: EK213
GSH Assay Kit	ABclonal Technology (China)	Catalog #RK05819
GST Assay Kit	Bionmkd (China)	Catalog #NMW0427
GSS Assay Kit	Bioesn (China)	Catalog #BES4809K
Cell Counting Kit-8	Beyotime (China)	Catalog #C0039
Tissue Mitochondria Isolation Kit	Beyotime (China)	Catalog #C3606
LDH Cytotoxicity Assay Kit	Beyotime (China)	Catalog #C0017
Lipid Peroxidation MDA Assay Kit	Beyotime (China)	Catalog #S0131S
Antioxidant Assay Kit	Sigma (USA)	Catalog #MAK334
Myco-Lumi™ Luminescent Mycoplasma Detection Kit	Beyotime (China)	Catalog #C0298S
Toluene-2,4-diisocyanate	Sigma-Aldrich (USA)	Catalog #89870

MitoSOX Red Mitochondrial Superoxide Indicator	YEASEN (China)	Catalog #40778ES50
Seahorse XF Cell Mito Stress Test Kit	Agilent (USA)	Catalog #103010-100
HiScript® IV All-in-one RT SuperMix Perfect for qPCR kits	Vazyme (China)	Catalog #R433-01
Total RNA Extraction Reagent	Vazyme (China)	Catalog #R401-01
ChamQ Universal SYBR qPCR Master Mix	Vazyme (China)	Catalog #Q711-02/03
House dust mite	Greer Labs (USA)	Catalog #421175
PneumaCult™-ALI Maintenance Medium	StemCell Technologies (Canada)	Catalog #05001
PneumaCult™-Ex Plus Medium	StemCell Technologies (Canada)	Catalog #05040
Discovery Studio 2023	BIOVIA (France)	
AutoDock Vina v1.12	https://vina.scripps.edu/	
SPSS 26.0	IBM (USA)	
Image J v1.53	National Institutes of Health	
electron microscope JEM-1010	JEOL (JAPAN)	
RNA-squencing data		GSE76262, GSE63142
