# Supplementary material for: Nrp1 Signaling Reprograms Glutathione Metabolism to Drive Mitochondrial Dysfunction in Severe Asthma
Source: Antioxidants (Basel). 2026 Apr 8;15(4):463. doi: 10.3390/antiox15040463 (PMC13114205; doi:10.3390/antiox15040463)
Supplement: Supplementary file 1 [file antioxidants-15-00463-s001.zip › Supplemental methods.pdf]

Supplemental materials

1. CatBoost model construction

A CatBoost-based machine learning model was constructed to evaluate the discriminative performance of candidate biomarkers for severe asthma. Prior to model development, the dataset was randomly shuffled to minimize potential sampling bias. The entire dataset was then randomly divided into a training set (70%) and a testing set (30%). To optimize model robustness and reduce overfitting, ten-fold cross-validation was performed on the training set during model development. Specifically, the training data were partitioned into ten subsets, with nine subsets used for training and one subset used for validation in an iterative manner. Hyperparameters were tuned based on cross-validation performance. The final model was subsequently evaluated on the independent testing set. Model performance was assessed using receiver operating characteristic (ROC) curve analysis, and the area under the ROC curve (AUC) was calculated to quantify the predictive accuracy. In addition, feature importance analysis was conducted based on the CatBoost algorithm to estimate the relative contribution of each biomarker to the classification task, thereby identifying key predictive genes associated with severe asthma.

2. Cellular thermal shift assay (CETSA)

The cellular thermal shift assay (CETSA) was performed to evaluate the direct binding of compounds to Nrp1 by assessing ligand-induced thermal stabilization of the target protein. Cells were treated with olopatadine or dabrafenib, or vehicle control, under the indicated conditions. Following treatment, cells were harvested and resuspended in PBS, then aliquoted into multiple tubes and exposed to a temperature gradient (43, 46, 49, 52, 55, 58, 61, 64, 67, and 70 °C) for 3 min using a thermal cycler. After heating, samples were immediately cooled to room temperature to allow protein aggregation. Cells were subsequently lysed by repeated freeze–thaw cycles, and the soluble protein fraction was separated by centrifugation at 12,000 × g for 10 min at 4 °C. The supernatants containing thermally stable proteins were collected and subjected to SDS–PAGE followed by immunoblotting analysis. The protein levels of Nrp1 were detected using a specific antibody, and changes in thermal stability were evaluated by comparing band intensities across different temperature points. Ligand-induced stabilization of Nrp1 was inferred from a shift in the thermal denaturation profile relative to the vehicle control, indicating potential direct interaction between the compounds and Nrp1.

3. Analysis of GEO data

All datasets are sourced from the GEO database, with the downloaded data in MINiML format, which includes all platforms, samples, and complete GSE records in the GSE. For datasets that have not been normalized, we uniformly perform log2 transformation. If the dataset has not been standardized, we use the normalize.quantiles function in the preprocess Core package of R for data standardization. According to the corresponding platform annotation information, we convert probe IDs into gene symbols, exclude probes corresponding to multiple genes, and calculate the average value of genes corresponding to multiple probes. For different batches within the same dataset and platform, we use the removeBatchEffect function in the limma package of R to remove

batch effects. When conducting a combined analysis of different datasets or data from different platforms within the same dataset, we first extract the common gene symbols from multiple datasets, then mark different datasets or different platforms as different batches, and also use the removeBatchEffect function to remove batch effects. Statistical analysis was conducted using R software, version v4.0.3. Results were considered statistically significant when the p-value was less than 0.05.

4. Plasmid construction and overexpression of SLC25A39

The SLC25A39 overexpression plasmid was constructed by cloning the full-length coding sequence of human SLC25A39 (NM_001143780, 1077 bp) into the pCMV expression vector containing a C-terminal 3×Flag-GFP tag (pCMV-SLC25A39-3Flag-GFP). The recombinant plasmid was synthesized and verified by a commercial provider (Guangzhou Dahong Biotechnology Co., Ltd., China). The construct confers kanamycin resistance for bacterial selection. For plasmid amplification, the recombinant plasmid was first transformed into DH5α competent Escherichia coli. Briefly, 2 μL of plasmid DNA was added to 100 μL of competent cells and incubated on ice for 30 min, followed by heat shock at 42 °C for 90 s. After recovery in antibiotic-free LB medium at 37 °C for 1 h with shaking, the bacteria were plated onto LB agar plates containing kanamycin and incubated overnight at 37 °C. Single colonies were picked and cultured in LB medium with kanamycin for 12–16 h. Plasmid DNA was then extracted using a standard plasmid purification kit and verified by PCR and sequencing prior to downstream applications. For functional studies, purified plasmids were transfected into target cells using a suitable transfection reagent according to the manufacturer’s instructions. Cells transfected with the empty vector served as controls. Overexpression efficiency was confirmed by immunoblotting using anti-Flag or anti-SLC25A39 antibodies, as well as by GFP fluorescence observation where applicable. A high-resolution version of the plasmid construction schematic is provided below in this document.

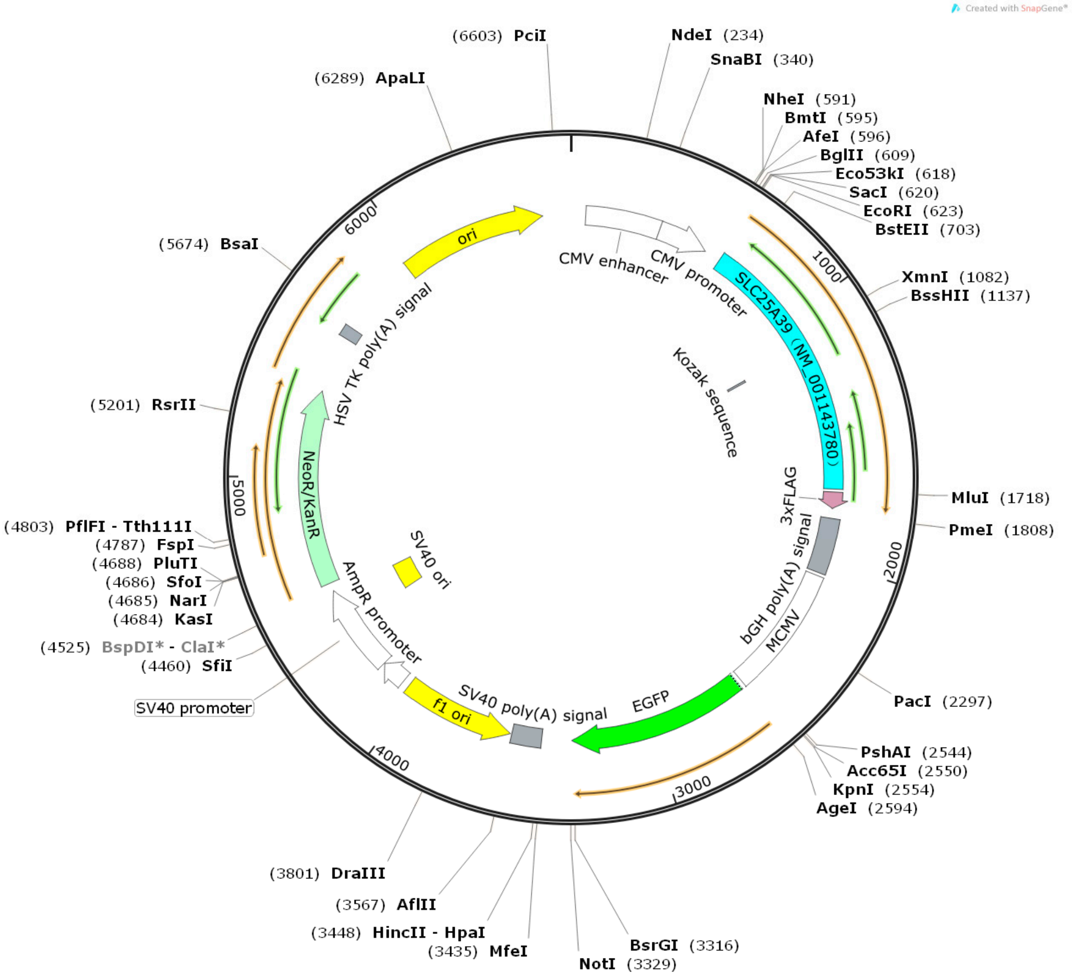

HY24598 pCMV-SLC25A39（NM_001143780）-3flag-gfp

6661 bp
