# Supplementary material for: Nrp1 Signaling Reprograms Glutathione Metabolism to Drive Mitochondrial Dysfunction in Severe Asthma
Source: Antioxidants (Basel). 2026 Apr 8;15(4):463. doi: 10.3390/antiox15040463 (PMC13114205; doi:10.3390/antiox15040463)
Supplement: Supplementary file 1 [file antioxidants-15-00463-s001.zip › Tables S1 to S7.pdf]

Table S1. Nucleotide sequences of the primers used for PCR

Gene	Primer Sequence 5' to 3'	
	**Forward**	**Reverse**
Neuropilin-1-Mouse	GACAAATGTGGCGGGACCATA	TGGATTAGCCATTCACACTTCTC
MMP-2-Mouse	TGCAGGAGACAAGTTCTGGAG	GTAGCTATGACCACCACCCTG
MMP-9-Mouse	CGTGTCTGGAGATTCGACTTGA	TGGTTCACCTCATGGTCCAC
VEGF-Mouse	CACTGGACCCTGGCTTTACT	ACTTGATCACTTCATGGGACTTCT
TGFβ1-Mouse	ACGTCACTGGAGTTGTACGG	GGGGCTGATCCCGTTGATT
Cytb-Mouse	CCCACCCCATATTAAACCCG	GAGGTATGAAGGAAAGGTATTAGGG
ATP6-Mouse	TCCCAATCGTTGTAGCCATC	TGTTGGAAAGAATGGAGACGG
COX1-Mouse	CCCAGATATAGCATTCCCACG	ACTGTTCATCCTGTTCCTGC
COX2-Mouse	AGTTGATAACCGAGTCGTTCTG	CTGTTGCTTGATTTAGTCGGC
IL-1β-Human	CTGTCCTGCGTGTTGAAAGA	TTGGGTAATTTTTGGGATCTACA
IL-25-Human	CCAGGTGGTTGCATTCTTGG	TGGCTGTAGGTGTGGGTTCC
IL-33-Human	CAAAGAAGTTTGCCCCATGT	AAGGCAAAGCACTCCACAGT
TSLP-Human	ATGTTCGCCATGAAAACTAAGGC	GCGACGCCACAATCCTTGTA
GAPDH-Mouse	AAGAGGGATGCTGCCCTTAC	CCAATACGGCCAAATCCGTTC
GADPH-Human	AGAAGGCTGGGGCTCATTTG	GGGGCCATCCACAGTCTTC
SLC25A39-Human	CCCTGGAGCTTATGCGGAC	GCCTGAACCCATTGAGCCA
E-cadherin-Human	CGAGAGCTACACGTTCACGG	GGGTGTCGAGGGAAAAATAGG
Claudin-1-Human	CCTCCTGGGAGTGATAGCAAT	GGCAACTAAAATAGCCAGACCT
Occludin-Human	GACTTCAGGCAGCCTCGTTAC	GCCAGTTGTGTAGTCTGTCTCA

Table S2. Correlation analysis between sputum GSHr and clinical features

V1	V2	Correlation	p_value
GSHr	ACT	0.358**	< 0.001
GSHr	FENO	-0.234*	0.010
GSHr	FEV_1/FVC, %	0.187*	0.040
GSHr	PEF	0.254**	0.005
GSHr	Neutrophils count, %	-0.333**	< 0.001

FEV1, forced expiratory volume in 1 s; FVC, forced vital capacity; PEF, peak expiratory flow; FeNO, Fractional exhaled nitric oxide; MEF, maximal expiratory flow.

Table S3. Predictive values of sputum GSH for predicting severe asthmatic patients

Test variables	AUC	Cutoff value	Sensitivity	Specificity	PPV	NPV	Youden index	P-value
GSHr (pg/mL)	0.860 (95% CI 0.80-0.92)	17.12	0.739	0.840	0.74	0.84	0.579	P<0.001

AUC, area under the curve; PPV, positive predictive values; NPV, negative predictive values; N.A., Not applicable.

Table S4. Predictive values of differential genes for predicting severe asthma patients

Test variables	AUC	Cutoff value	Sensitivity	Specificity	PPV	NPV	Youden index	P-value
GSTP1	0.764 (95% CI 0.68-0.85)	9.202	0.516	0.891	0.90	0.47	0.407	P < 0.001
GSTK1	0.815 (95% CI 0.74-0.89)	9.050	0.796	0.783	0.88	0.64	0.579	P < 0.001
MGST2	0.794 (95% CI 0.72-0.87)	8.317	0.731	0.761	0.86	0.58	0.492	P < 0.001
MGST3	0.772 (95% CI 0.70-0.85)	9.198	0.473	0.978	0.98	0.48	0.451	P < 0.001
GSTO1	0.731 (95% CI 0.68-0.85)	10.830	0.667	0.696	0.82	0.51	0.363	P < 0.001
MGST1	0.616 (95% CI 0.52-0.71)	7.071	0.312	0.935	0.91	0.40	0.247	P = 0.026
GSTT1	0.694 (95% CI 0.59-0.80)	7.022	0.849	0.565	0.80	0.65	0.414	P < 0.001
GSS	0.744 (95% CI 0.66-0.83)	8.423	0.742	0.696	0.83	0.57	0.438	P < 0.001
SLC25A39	0.658 (95% CI 0.56-0.76)	6.356	0.710	0.609	0.79	0.51	0.319	P < 0.001
AFG3L2	0.715 (95% CI 0.63-0.80)	7.070	0.591	0.848	0.89	0.51	0.439	P < 0.001

AUC, area under the curve; PPV, positive predictive values; NPV, negative predictive values; N.A., Not applicable.

Table S5. Predictive values of differential genes for predicting severe asthma patients

Test variables	AUC	Cutoff value	Sensitivity	Specificity	PPV	NPV	Youden index	P-value
GSTA1	0.675 (95% CI 0.58-0.77)	11.013	0.714	0.616	0.51	0.79	0.330	P < 0.001
GSTA2	0.687 (95% CI 0.61-0.75)	12.421	0.768	0.556	0.49	0.81	0.324	P < 0.001
GSTA3	0.690 (95% CI 0.60-0.78)	10.658	0.679	0.687	0.55	0.79	0.366	P < 0.001
GSTA5	0.704 (95% CI 0.62-0.79)	11.389	0.679	0.697	0.56	0.79	0.376	P < 0.001
GSTM2	0.609 (95% CI 0.52-0.70)	8.000	0.661	0.616	0.49	0.76	0.277	P = 0.024

AUC, area under the curve; PPV, positive predictive values; NPV, negative predictive values; N.A., Not applicable.

Table S6. The docking score and predicted protein–ligand interaction of the top eight compounds selected in virtual screening

Compound Name	Molecular Formula	Weight (g/mol)	LibDock Score	AutoDock Score	Noncovalent Interactions	Residues
Olopatadine	$C_{21}H_{23}NO_3$	337.41	114	-7.8	2 H-bond, 2 pi-Pi-bond	ASP320, THR349, TYR353, SER346
Ndga	$C_{18}H_{22}O_4$	302.36	115	-8	3 H-bond, 2 pi-Pi-bond	ASP320, TYR353, ILE146
Oxatomide	$C_{27}H_{30}N_4O$	426.55	125	-8.1	1 H-bond, 1 pi-Pi-bond, 2 Pi-Anion bond	ASP320, THR349, TYR353
4-Hydroxy Ketorolac	$C_{15}H_9D_4NO_4$	275.29	107	-7.8	4 H-bond, 1 Pi-Anion bond	ASP320, THR349, TYR353, SER346
Lorazepam Glucuronide	$C_{21}H_{18}C_{l2}N_2O_8$	497.28	112	-8	2 H-bond, 1 pi-Pi-bond, 1 unfavorable positive-positive bond	ASP320, THR349, TYR353
Oxazepam Glucuronide	$C_{21}H_{19}ClN_2O_8$	462.84	129	-8	3 H-bond, 1 pi-Pi-bond, 1 unfavorable positive-positive bond	ASP320, THR349, TYR353
Dabrafenib	$C_{23}H_{20}F_3N_5O_2S_2$	519.56	105	-8.5	4 H-bond, 1 pi-Pi-bond, 1 Pi-Anion bond	ASP320, THR349, TYR353, SER346

Table S7. Binding free energies predicted using MMPBSA approach for Nrp1's b1 domain complexed with different compounds

Complex	Energy (Kcal/mol)						
	ΔVDWAALS	*ΔE* ELE	Δ*E* GB	Δ*E* SURF	Δ*G* GAS	Δ*G* SOLV	ΔTotal
Olopatadine	-40.77	-30.57	40.70	-5.28	-71.34	35.42	-35.91
Dabrafenib	-22.23	-56.88	57.00	-3.65	-79.11	53.36	-25.75

VDWAALS, van der Waals energy. ELE, electrostatic energy. GB, polar solvation energy (Generalized Born). SURF, non-polar solvation energy (SASA model). GAS, gas phase energy. SOLV, solvation free energy. Total, total binding free energy.
