# Supplementary material for: Nrp1 Signaling Reprograms Glutathione Metabolism to Drive Mitochondrial Dysfunction in Severe Asthma
Source: Antioxidants (Basel). 2026 Apr 8;15(4):463. doi: 10.3390/antiox15040463 (PMC13114205; doi:10.3390/antiox15040463)
Supplement: Supplementary file 1 [file antioxidants-15-00463-s001.zip › Figures S1 to S8.pdf]

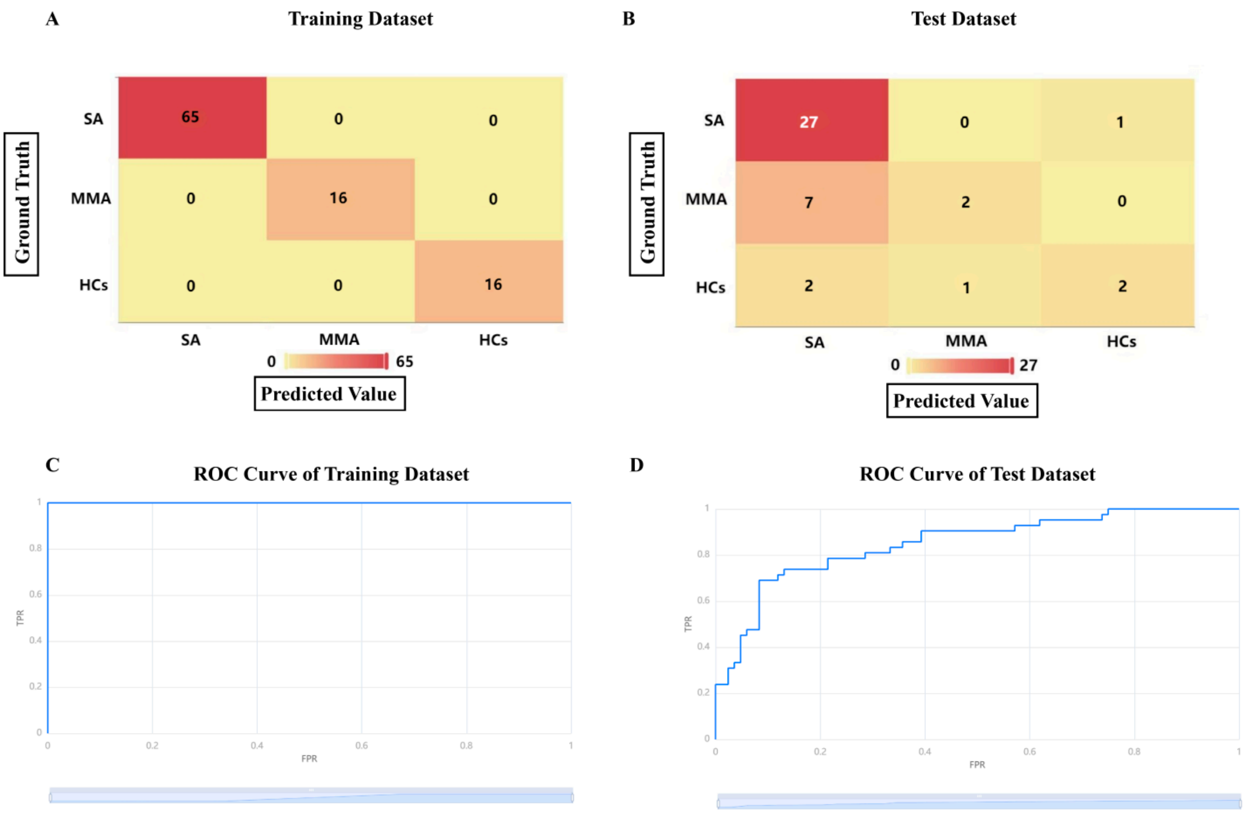

Figure S1. CatBoost model of GSH-metabolizing enzymes for diagnosis

(A) Confusion matrix heatmap of training dataset. (B) Confusion matrix heatmap of test dataset. (C) ROC curve of training dataset for diagnosis of HCs, MMA and SA. (D) ROC curve of test dataset for diagnosis of HCs, MMA and SA.

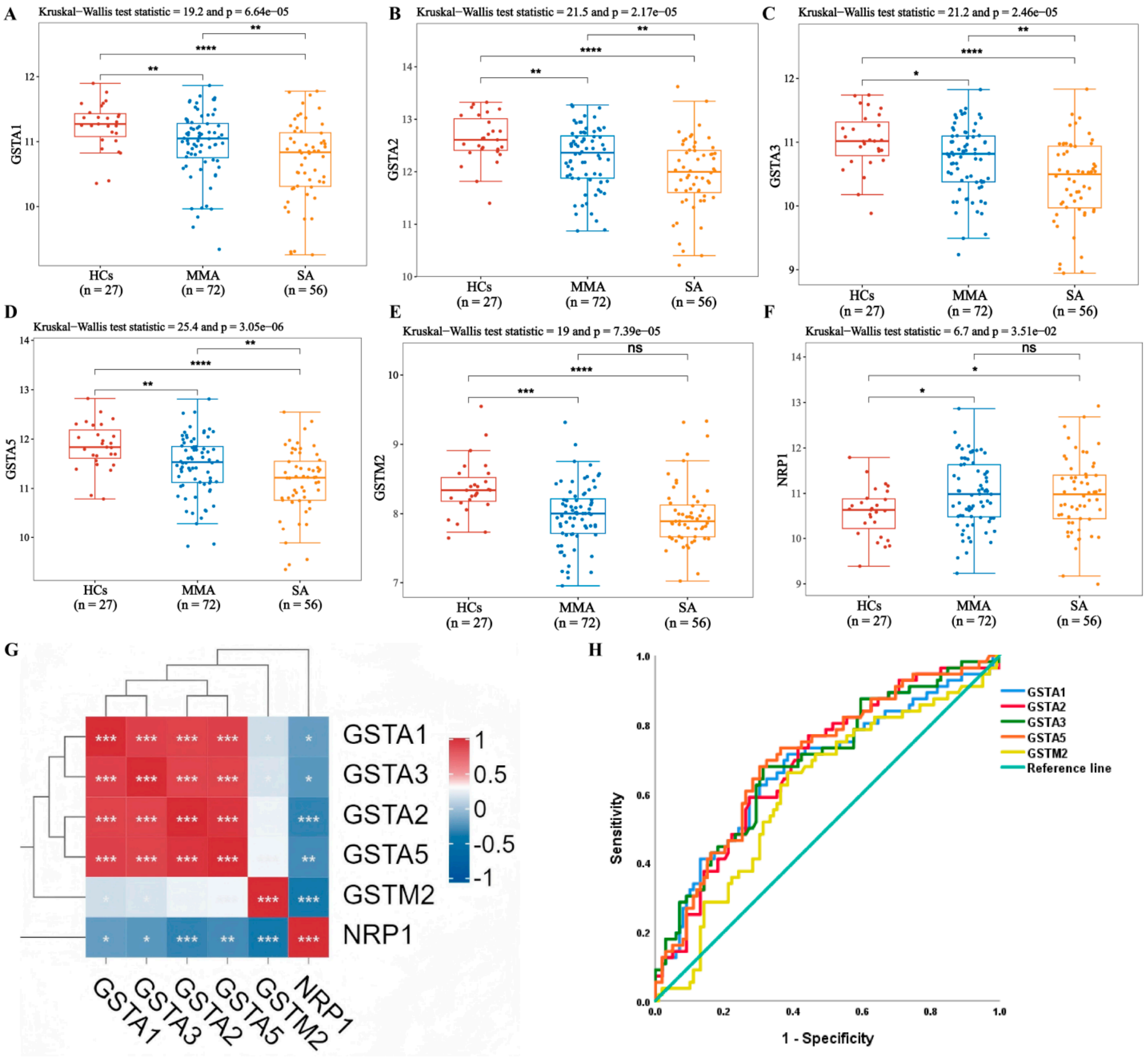

Figure S2. Human airway epithelial biopsy transcriptomic analysis

(A-F) Expression levels of GSTA1, GSTA2, GSTA3, GSTA5, GSTM2 and NRP1of transcriptomics. (J) Correlation analysis between Nrp1 and GSH-metabolizing enzymes. (K) ROC curve for GSH-metabolizing enzymes in predicting the diagnosis of severe asthma. *P < 0.05, **P < 0.005, ***P < 0.0005, ns > 0.05.

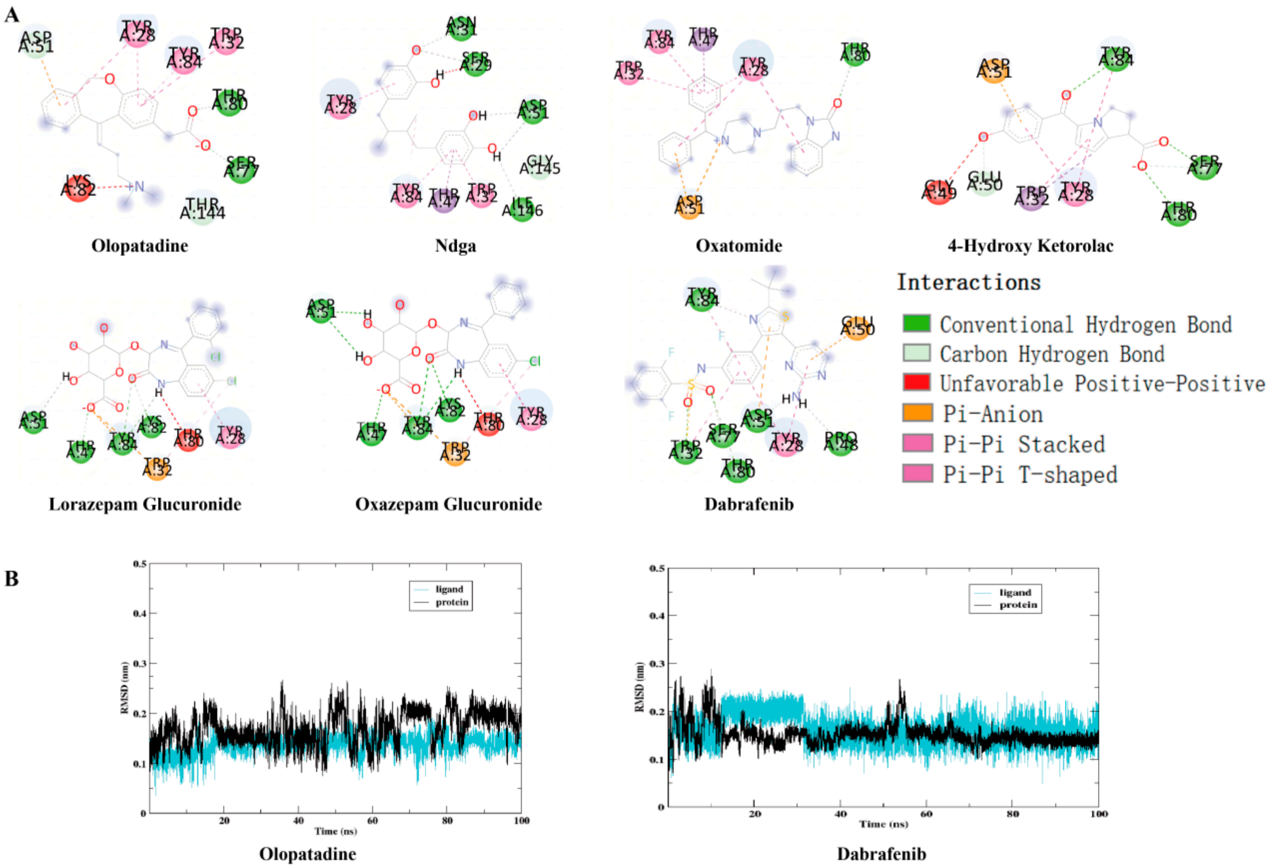

Figure S3. Ligands binding the b1 domain of Nrp1

(A) The predicted binding mode between the b1 domain of Nrp1 and ligands. The protein–ligand interactions of representative docking poses of the top seven hits are displayed around. (B) The RMSD plot over time of Nrp1's b1 domain and the compounds, displays the changes in the spatial structure of Nrp1's b1 domain and the ligands over 100ns, highlighting any deviations from its original conformation.

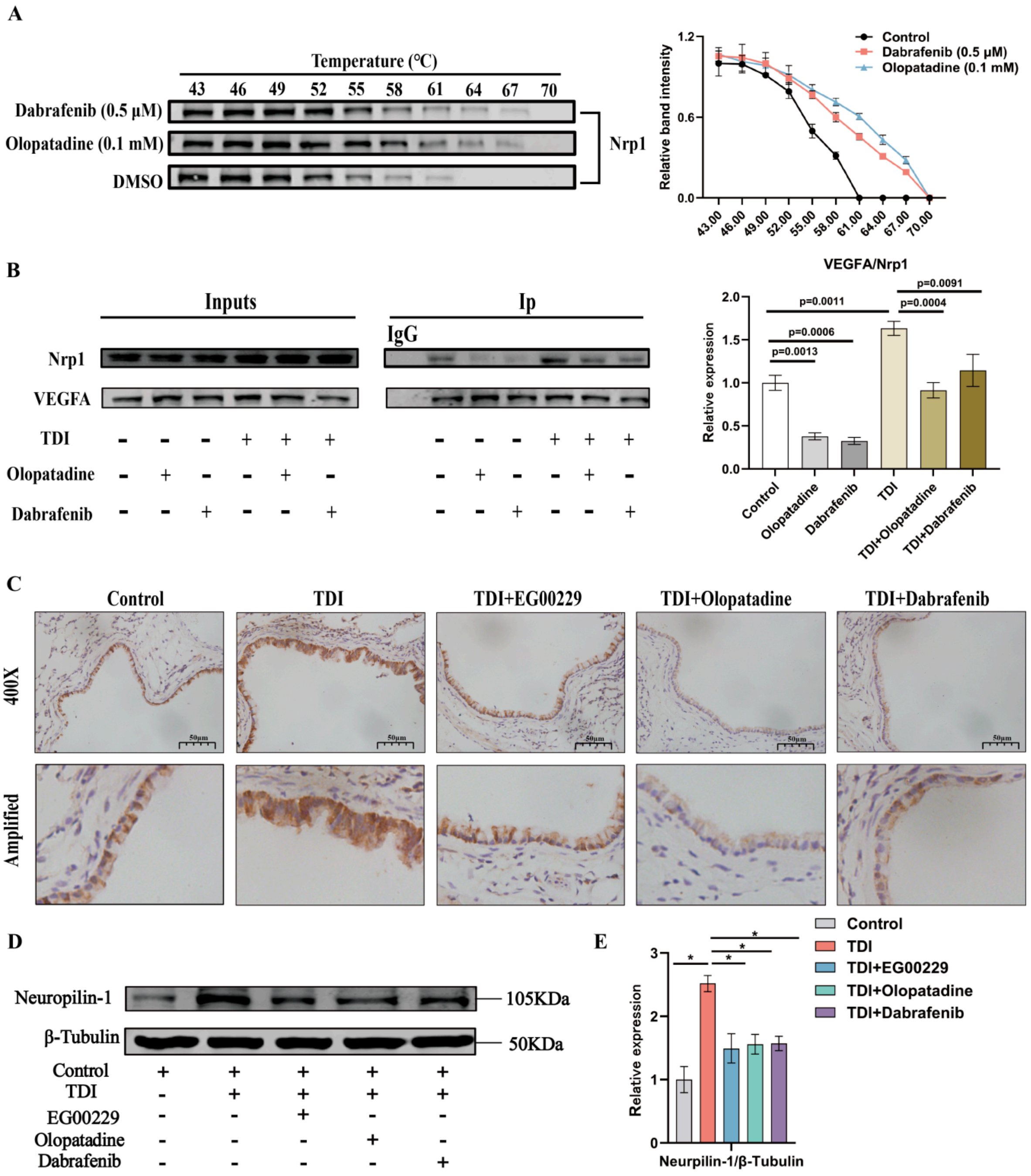

Figure S4. Olopatadine and dabrafenib inhibited the the protein binding levels of Nrp1 and VEGFA

(A) The protein expression of Nrf2 of Nrp1. (B) Co-immunoprecipitation between Nrp1 and VEGF of murine lung tissues were analyzed by western blot. (C) Immunohistochemistry analysis of Nrp1 in the airway. (D-E) Expression levels of Nrp1 of murine lung tissues. n=3. ***P < 0.0005.

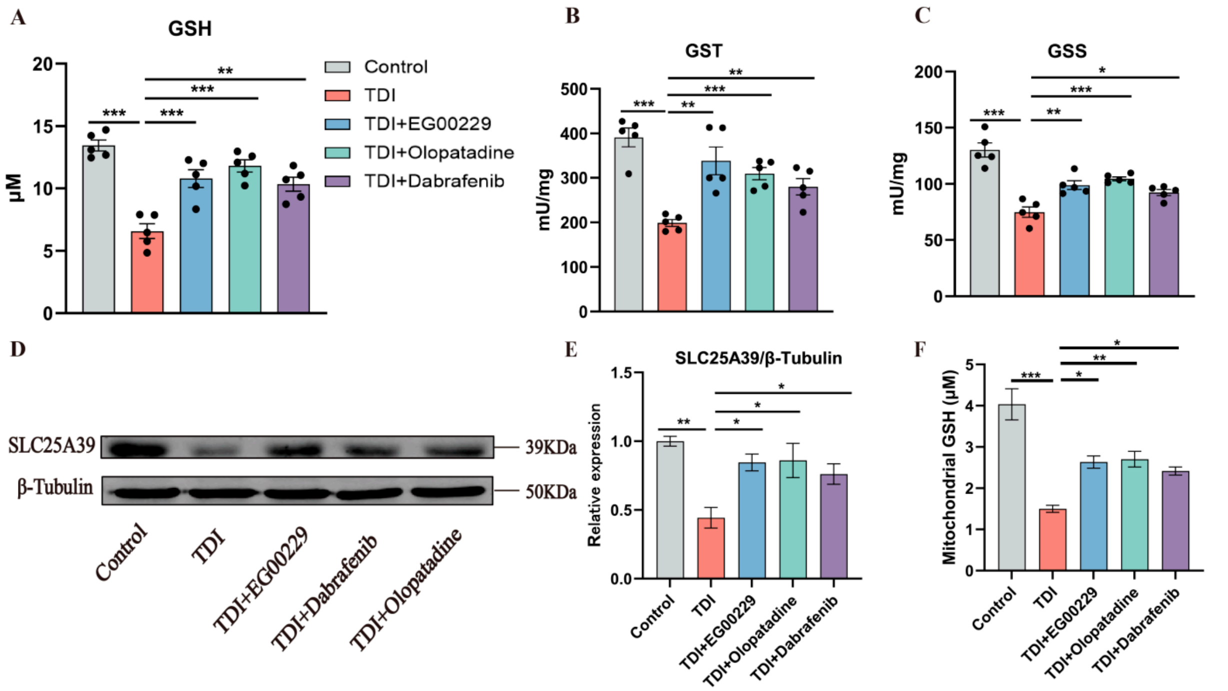

Figure S5. Nrp1 regulated GSH metabolism in the TDI-induced asthma model

(A-C) Detection of GSH, GST, GSS in the lung tissue of mice. (D-E) Western blotting of SLC25A39 and β-Tubulin expression in whole lung tissue. (F) Measurement of mitochondrial GSH. A mixture of 2 vol of acetone and 3 vol of olive oil was used as the vehicle (Control). ***P < 0.0005.

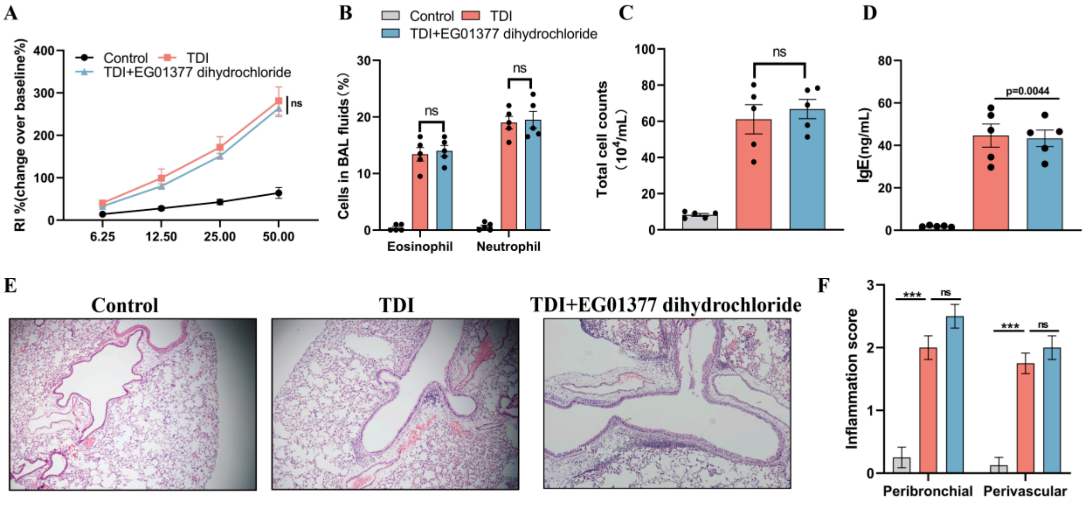

Figure S6. Effect of EG01377 dihydrochloride on TDI-induced airway inflammation

(A) AHR was measured by lungresistance (RL). Results were shown as percentage over baseline value (n=5). (B,C) Total and differential inflammatory cells in BALF (n=5). (D) Measurement of IgE in serum by ELISA (n=5). (E-F) Representative H&E stained lung sections of different groups at 100× original magnification. A mixture of 2 vol of acetone and 3 vol of olive oil was used as the vehicle (Control). ***P < 0.0005.

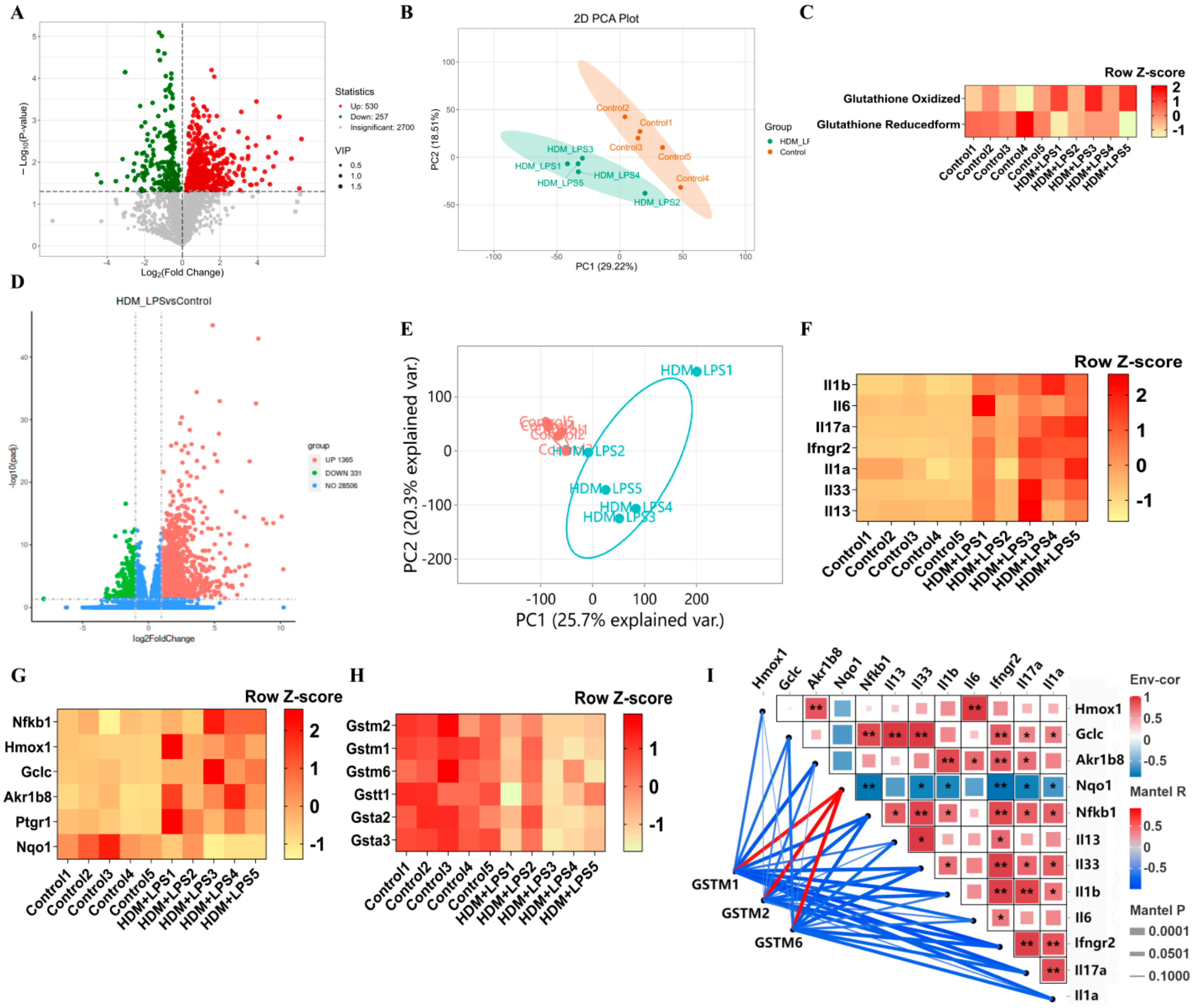

Figure S7. Dysregulation of GSH metabolism in the HDM/LPS-induced SA model

(A) Volcano analysis of metabolomics (HDM+LPS vs control). (B) Principal component analysis (PCA) of metabolomics. (C) Heatmap analysis of differential expressed metabolites. (D) Volcano analysis of transcriptomics (HDM+LPS vs control). (E) PCA of transcriptomics. (F-H) Heatmap analysis of

differential expressed genes. (I) Heat map of correlation analysis between differential expressed genes. *P < 0.05, **P < 0.005, ***P < 0.0005, ns > 0.05.

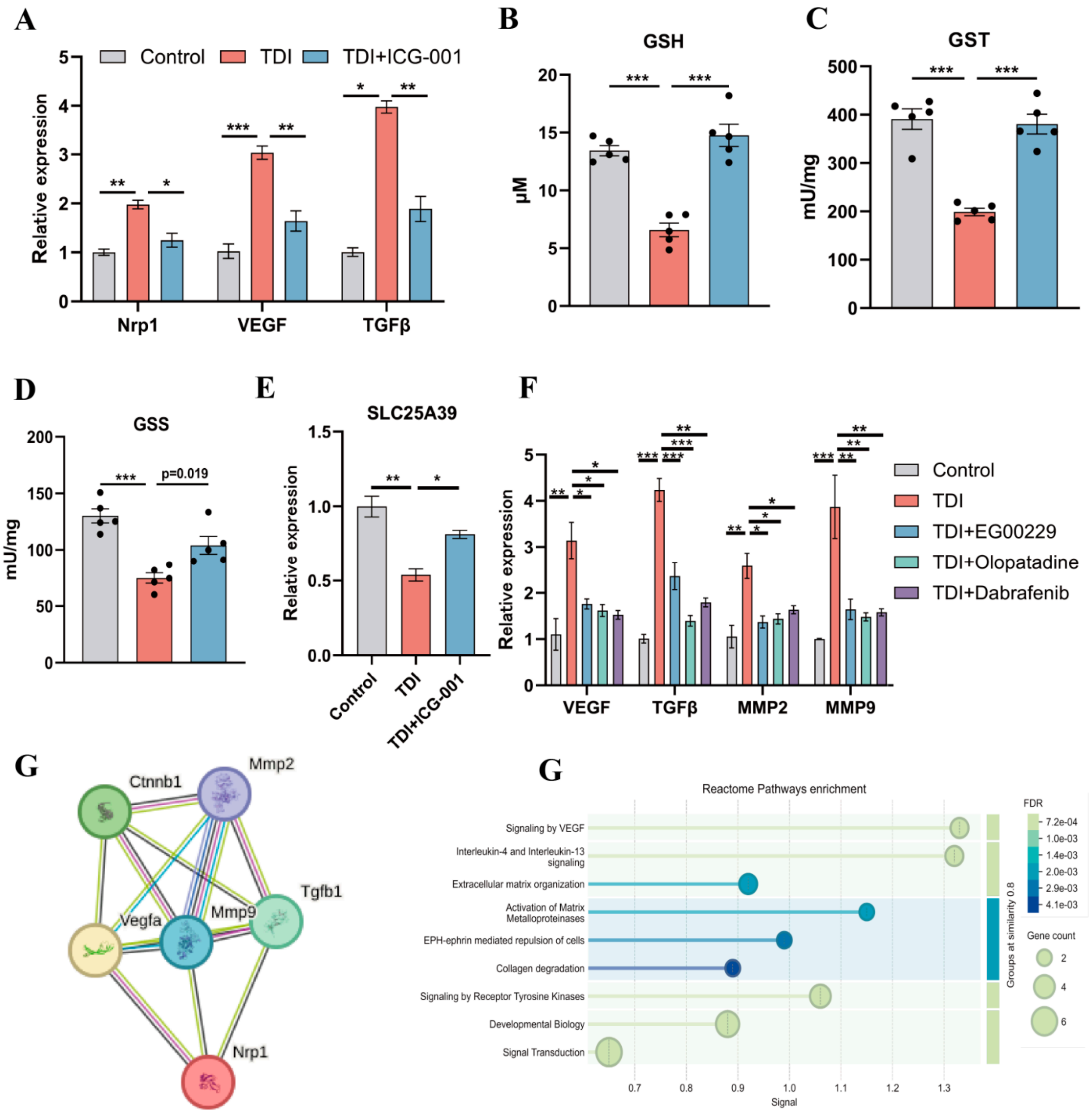

Figure S8. The mechanism of β-catenin-Nrp1 axis in regulating TDI-induced asthma model

(A) mRNA expression of Nrp1, VEGF, TGFβ in the whole lung. (B-D) Detection of GSH, GST, GSS in the lung tissue of mice. (E) mRNA expression of SLC25A39 in the whole lung. (F) mRNA expression of VEGF, TGFβ, MMP2, MMP9 in the whole lung. (G) Analysis of string network. *P < 0.0005, n=3-5.
